# Neuropeptide Diversity Encoded in Newly Sequenced Crustacean Genomes Reveals Signaling Roles during Feeding

**DOI:** 10.1021/acschemneuro.6c00123

**Published:** 2026-06-11

**Authors:** Lauren Fields, Vu Ngoc Huong Tran, Thao Duong, Tina C. Dang, Kendra G. Selby, Lingjun Li

**Affiliations:** 1 Department of Chemistry, 5228 University of Wisconsin, 1101 University Avenue,Madison, Wisconsin 53706, United States; 2 School of Pharmacy, University of Wisconsin, 777 Highland Avenue, Madison, Wisconsin 53705, United States; 3 Lachman Institute for Pharmaceutical Development, School of Pharmacy, University of Wisconsin-Madison, Madison, Wisconsin 53705, United States; 4 Wisconsin Center for NanoBioSystems, School of Pharmacy, University of Wisconsin-Madison, Madison, Wisconsin 53705, United States

**Keywords:** neuropeptide, genomics, mass spectrometry, *Cancer borealis*, *Callinectes sapidus*, endogenous

## Abstract

Neuropeptides are chemically diverse signaling molecules that regulate physiology and behavior, yet many species lack neuropeptidomic characterization due to sparse genomic annotation. Here, we integrate genomic sequence information with neuropeptidomics to define the neuropeptidomes of two widely used crustacean model organisms, *Callinectes sapidus* and *Cancer borealis*. Using a curated multispecies precursor database, tBLASTn alignment, signal peptide detection, and *in silico* processing, we predicted more than 23,000 putative peptides across both genomes, including numerous sequences bearing hallmarks of mature neuropeptides. Mass spectrometry-based profiling provided experimental support for many predicted peptides and revealed substantial chemical diversity, including novel allatostatin B and C isoforms, an insulin-like peptide B-chain-like isoform in *C. sapidus*, and the first report of natalisin peptides in *C. borealis*. Notably, we observed an atypical precursor architecture in which a single prohormone encoded two distinct neuropeptide families, suggesting previously unrecognized modes of neuropeptide copackaging and signaling. Finally, leveraging a feeding perturbation model, we observed tissue-specific differences in the abundance of newly identified peptides in the thoracic ganglion, commissural ganglion, and pericardial organs, consistent with functional neuroendocrine roles. Together, this work expands the known repertoire of crustacean neuropeptides, provides a resource for comparative peptide biology, and establishes a genome-enabled framework for discovery of endogenous peptide signaling molecules in newly sequenced species.

## Graphical abstract

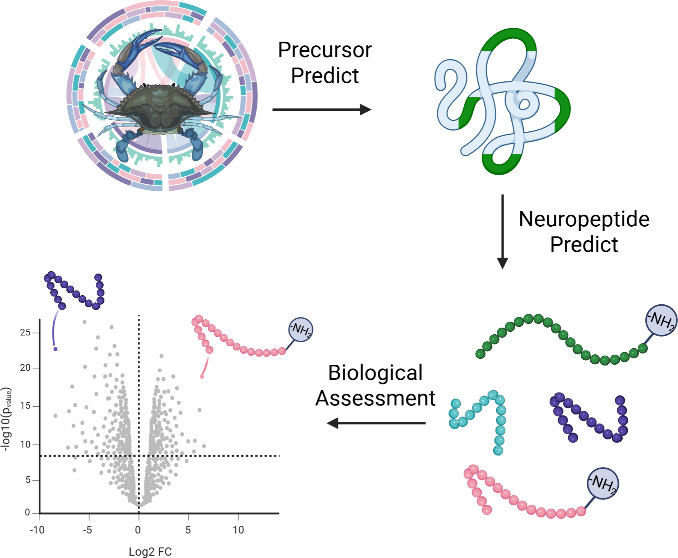

## Introduction

Neuropeptides are critical signaling molecules secreted by neurons that play essential roles in regulating physiology and have garnered wide attention in healthcare as both biomarkers and therapeutics. Insulin and glucagon-like peptide (GLP-1) peptides are renowned for their contributions to modern medicine and notably originate as prohormones that encode bioactive peptides.^1–3^ Despite their tremendous importance, much remains unknown about neuropeptides. This knowledge gap stems in part from the complexities of comodulation, in which two or more neuropeptides act together to achieve a shared function.^4^ One established approach for unraveling the intricate players behind these complex outcomes is to leverage invertebrate model organisms, which provide a simpler testbed for parsing regulatory functions and applying environmental or biological perturbations to delineate their impact on neuropeptide responses. While rats, mice, *Drosophila*, nematodes, and other organisms have been used in this arena, a model organism of particular prominence for neurochemical investigations is the crab.^5^ Its popularity can be attributed in part to the relative simplicity of the crustacean stomatogastric nervous system (STNS), which governs many homeostatic processes. For example, other simple model organisms, such as the nematode (*e.g.*, *C. elegans*) have approximately 300 neurons in their nervous system, whereas the stomatogastric ganglion of crustaceans such as *C. borealis*, have approximately 30 neurons,^6^ making them ideal models for neuropeptide research.

A variety of crustacean species have been used to elucidate neuropeptide-driven processes, and as a result, some of the most well-characterized neural circuits originate from these organisms.^7^ For example, the gastric mill and pyloric rhythm, central to feeding behavior, are most robustly characterized in the Jonah crab, *Cancer borealis*.^8,9^ Additionally, *Callinectes sapidus*, or the blue crab, has been extensively used to study a variety of environmental-based stressors,^10^ including hypoxia,^11–13^ temperature sensitivity,^10^ pH perturbations,^14^ and more.^15^ Despite this wealth of knowledge, progress in this area has historically been limited due to the lack of genome assemblies. Two of the most widely used crustacean model systems, *C. sapidus* and *C. borealis*, were only recently assembled and published in 2021 and 2024, respectively.^16,17^ In the interim, neuropeptidomic insights have been gained through *in silico* prediction from transcriptomics, experimental methods such as *de novo* sequencing by mass spectrometry (MS), and related approaches. However, genome-level information provides unprecedented access to the neuropeptidome. Neuropeptides are highly specialized signaling molecules, with high sequence similarity among peptides that perform distinct functional roles. Thus, although Edman degradation and later *de novo* sequencing via MS have helped characterize neuropeptide sequences, these methods are restricted to peptides in their processed forms.^18^ Transcriptomics has also been heavily applied; however, genome-level analysis provides complementary and independent sequence information, enabling detection of variants that may not be represented in available transcriptomic data sets, particularly for species with limited transcriptomic coverage.^19–25^ For example, the most comprehensive *C. borealis* transcriptome-based neuropeptidome to date was derived from pooled nervous system tissues,^26^ and would overlook peptides expressed primarily in the peripheral neuroendocrine organs such as the pericardial organs or the sinus glands. Genome-based prediction is tissue- and condition-agnostic, providing access to the full peptide encoding potential of the organism independent of sampling decisions.

With the emergence of more sophisticated prediction models, we sought to use a genome-driven *in silico* workflow to predict the neuropeptidomes of *Callinectes sapidus* (blue crab) and *Cancer borealis* (Jonah crab) from their recently published genomes. Our findings revealed several previously unreported neuropeptides, including novel isoforms and an atypical precursor architecture encoding two distinct neuropeptide families. Moreover, this work demonstrates that by integrating genome-based *in silico* predictions with existing transcriptome-derived predictions and data from *de novo* sequencing and other experimental methods, we can generate a more comprehensive representation of the neuropeptidome. This expanded landscape included notable discoveries, such as insulin-like peptide (ILP) isoforms and a single neuropeptide precursor encoding two distinct neuropeptide families.

Finally, we applied our findings to one of the most established biological contexts for Jonah crabs: feeding. We evaluated neuroendocrine tissues postfeeding using our newly curated *in silico* peptide database and used these predictions to assess patterns in neuropeptide abundance across tissues. In particular, we observed several neuropeptides displaying differential abundance between the thoracic ganglion (TG) and pericardial organs (POs), consistent with tissue-specific neuroendocrine roles.

## Results and Discussion

To elucidate neuropeptides from the genome, we first compiled a database spanning a wide variety of neuropeptide families from diverse crustacean species previously reported in UniProt (Supplemental File 1). To ensure broad coverage, we included searches explicitly targeting key neuropeptide families (Figure 1A). Approximately 40% of the precursor sequences used as queries were classified as level 4 according to UniProt, representing predicted sequences (Figure 1B). Another 50% of the precursors came from levels 2 or 3, indicating moderate evidence, representing transcript-level evidence and putative identification based on homology, respectively. Just 10% of precursors reflected the highest level of confidence with protein-level evidence (level 1). Finally, upon further inspection, approximately half of the queries corresponded to true peptides, including fully processed mature peptides (‘peptide’), sequences with peptide-like features but uncertain processing (‘peptide-like’), partial peptide sequences (‘peptide fragment’), or peptides mapping to the flanking regions of a precursor (‘precursor-related peptide’). The remaining sequences were classified as precursors, including confirmed precursors, isoforms, or truncated precursor sequences (Figure 1C). After obtaining precursor sequences, we aligned them to the *C. sapidus* and *C. borealis* genomes using tBLASTn, identifying genomic regions with high similarity to known precursors (Figure 1D). As expected, most database entries originated from shrimp and crab species, reflecting the substantial body of crustacean neuropeptide research in the literature (Figure 1E). A detailed list of organisms included in the search is provided in Figure S1. The resulting genomic hits were then translated and evaluated for the presence of a signal peptide region. Because signal peptides are a defining feature of neuropeptide precursors, only sequences containing a putative signal peptide were retained for downstream analysis. Once signal peptides were identified, putative neuropeptides were predicted by inducing cleavage at basic residues or at sites directly following the signal peptide via NeuroPred.^27^

**Figure 1:**
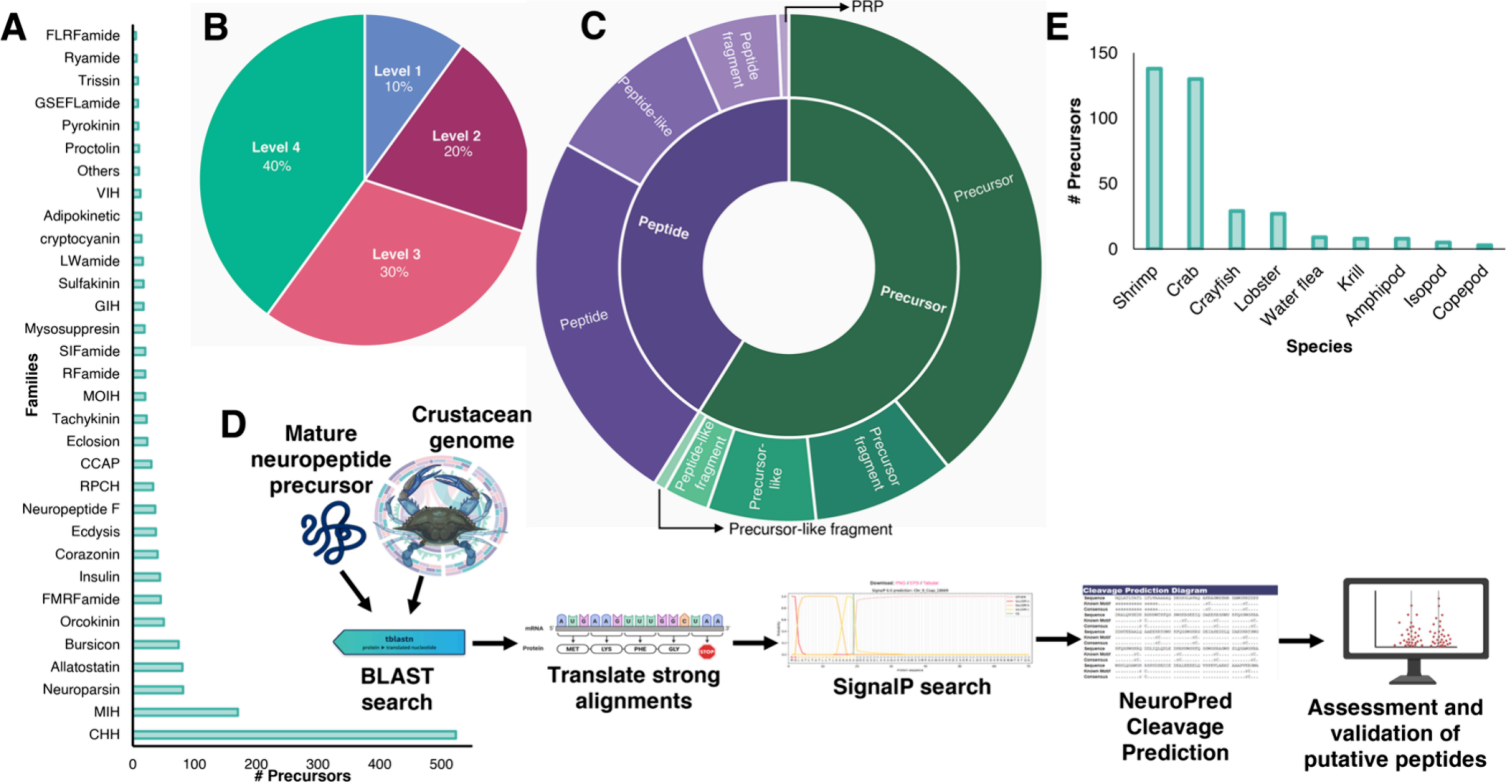
Distribution of precursors used for *in silico* prediction of neuropeptides. **A)** Number of entries per neuropeptide family. **B)** Level of UniProt documentation for the entry. Level 1 represents the most confident identifications, with support at the protein level. Subsequent levels 2, 3, and 4 represent decreasing confidence, representing support at the transcript level, homology-based identification, and predicted protein. Level 5 (protein uncertain) were omitted from this analysis. **C)** True query classifications as peptide or precursor, with additional delineation. **D)** General workflow for prediction. **E)** Organisms used for prediction. Abbreviations: vitellogenesis inhibiting hormone (VIH), gonad inhibiting hormone (GIH), mandibular organ-inhibiting hormone (MOIH), crustacean cardioactive peptide (CCAP), red pigment concentrating hormone (RPCH), molt-inhibiting hormone (MIH), crustacean hyperglycemic hormone (CHH), precursor-related peptide (PRP).

### Prediction of Putative Neuropeptides

Applying this workflow, we identified 14,569 potential peptides for *C. borealis* and 8,892 peptides for *C. sapidus* (Figure S2). Predicted peptides for *C. borealis* and *C. sapidus* are detailed in Supplemental File 2 and Supplemental File 3, respectively. While this number is substantial, these peptides were derived from only 66 putative precursors in *C. sapidus*, for example. This relatively small number of precursor genes likely reflects the sparse annotation of both genomes rather than a true biological ceiling, as both assemblies were recently published and remain incompletely annotated. To evaluate the validity of these predictions, we first examined peptides for features indicative of maturity. In general, mature neuropeptides that undergo C-terminal amidation exhibit several hallmark characteristics, including an amidated C-terminus preceded by a glycine residue and a dibasic cleavage site.^28^ Additional maturity criteria, including motif conservation, were also considered to account for mature peptides that do not undergo C-terminal amidation.^6^ The mature peptides identified in *C. borealis and C. sapidus* are listed in Tables S1 and S2, respectively. Interestingly, we also identified several mature natalisin peptides, a notable finding given their conserved roles in arthropod neurobiology.^29^

To further contextualize these findings, we evaluated the topology of the identified natalisin peptides. All mature natalisin peptides were preceded by a signal peptide and mapped to the same precursor, which encoded a variety of amidated and nonamidated peptides (Figure 2A). The amidated peptides exhibited the expected pattern of glycine prior to the cleavage site (Table S3). Regarding signal peptides, the lengths predicted by SignalP for *C. sapidus* and *C. borealis* displayed a similar distribution, ranging from as few as 9 to as many as 49 residues (Figure 2B). Because basic residues are essential for proteolytic processing, we examined the identities of cleavage sites at the N-terminus (Figure 2C) and C-terminus (Figure 2D) of mature *C. borealis* peptides relative to their precursor sequences. While dibasic cleavage sites were most common, we also observed a handful of monobasic cleavages, which have been reported elsewhere.^30^ This was further supported by WebLogo analysis, where we evaluated the homology of peptides extending beyond the flanking basic residues illustrated in Figure 2 **C,D**. Interestingly, when plotting mature peptides, distinct conserved residues were most prominently visible in *C. borealis* (Figure S3A), where a glycine clearly dominates immediately upstream of the N-terminus cleavage site. Given the significance of glycine for C-terminal amidation of neuropeptides, it is possible that glycine is mechanistically important at the N-terminus as well. While more subtle, conservation was also observed in *C. sapidus* (Figure S3B), with potential motifs preceding the cleavage site.

**Figure 2:**
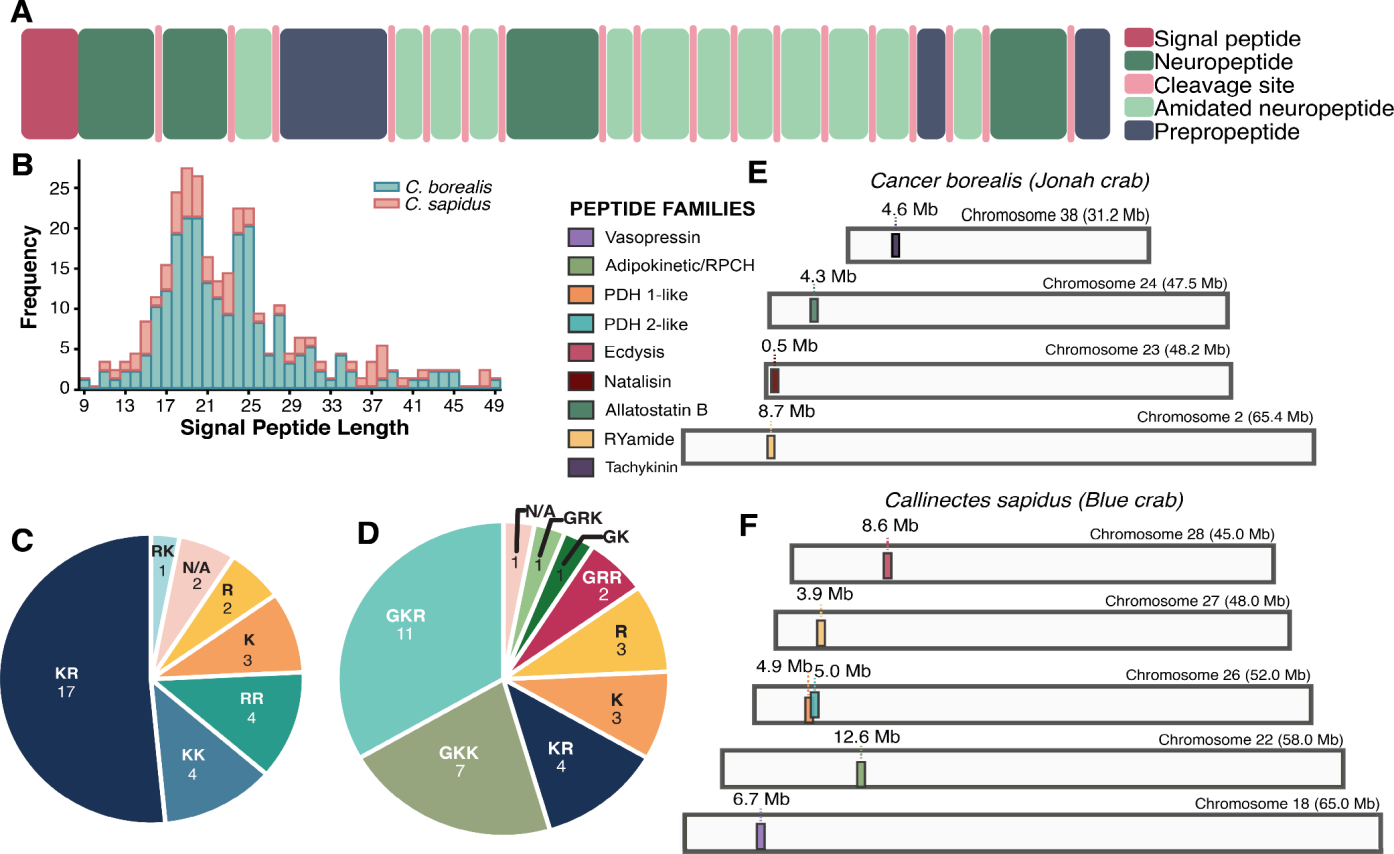
Mature neuropeptide attributes from findings. **A)** Precursor topography of a *C. borealis* natalisin precursor, annotated by color to denote the signal peptide, neuropeptide regions, cleavage sites, amidated neuropeptides, and prepropeptides. Classifications are based on annotation and inference from homologous precursor proteins reported in UniProt and the literature. In this context, ‘neuropeptide’ refers to the predicted mature peptide region, whereas ‘prepropeptide’ refers to the broader precursor-derived sequence context and does not imply lack of biological function. **B)** Distribution of signal peptide lengths for *C. borealis* and *C. sapidus* putative precursors. **C)** N-termini and **D)** C-termini sequence distribution for identified, confirmed peptides in *C. borealis*. “N/A” refers to terminal sequences lacking a basic residue. Chromosomal mapping of the mature precursors found in **E)** *Cancer borealis* and **F)** *Callinectes sapidus*. Abbreviations: red pigment concentrating hormone (RPCH), pigment dispersing hormone (PDH).

We next mapped these mature precursors back onto the genomes. In *C. borealis,* 36 neuropeptides mapped to four precursor genes, each located on a distinct chromosome (Figure 2E). Notably, the natalisin precursor encoded 16 peptides, while the allatostatin B precursor produced 18 peptides (Figure 2E). For *C. sapidus*, we mapped 16 mature neuropeptides to six genes across five chromosomes (Figure 2 **F)**. The most prolific family in the *C. sapidus* analysis was ecdysis-triggering hormone, supported by five mature neuropeptides (Table S4).

### Isoform Detection via Mass Spectrometry

To experimentally validate our *in silico* predictions, we performed MS analysis of *C. sapidus* and *C. borealis* to assess the biological context of these predicted peptides within their respective genomes. We then mapped experimentally identified peptides, obtained from database searching of MS results, back onto their corresponding precursors. Notably, these identifications provided additional evidence supporting the accuracy and relevance of our predicted neuropeptidome. For example, several novel peptides (*e.g.*, GAWGKR, FQGSWGKR) appeared repeatedly within the *C. borealis* precursor derived from transcript g30501. As is well-known, such repetition often hints at a conserved neuropeptide sequence. While these peptides are not deeply characterized as neuropeptides, we used their recurrence as a guide to annotate the precursor more comprehensively.

Using this approach, we also identified novel isoforms of known neuropeptides derived from the genome. For example, we detected several neuropeptides corresponding to the allatostatin B (AST-B) family. Although the identified fragments were relatively short, they highlighted conserved repeats that could be extrapolated to mature neuropeptides. For instance, the peptide QGSWGKR appeared four times within the precursor (Figure 3A). The QGSW sequence clearly represented the C-terminal end of the peptide, where the -GKR was consistent with peptide amidation followed by a dibasic cleavage site. By extending this sequence upstream toward the preceding dibasic cleavage site (*i.e.,* KR), several known mature peptides including NNWSKFQGSWamide,^31–34^ TSWGKFQGSWamide,^4,31–33,35^ NNNWSKFQGSWamide^33,35,36^ and GGWNKFQGSWamide,^35,37^ were readily apparent, all of which have previously been reported.

**Figure 3:**
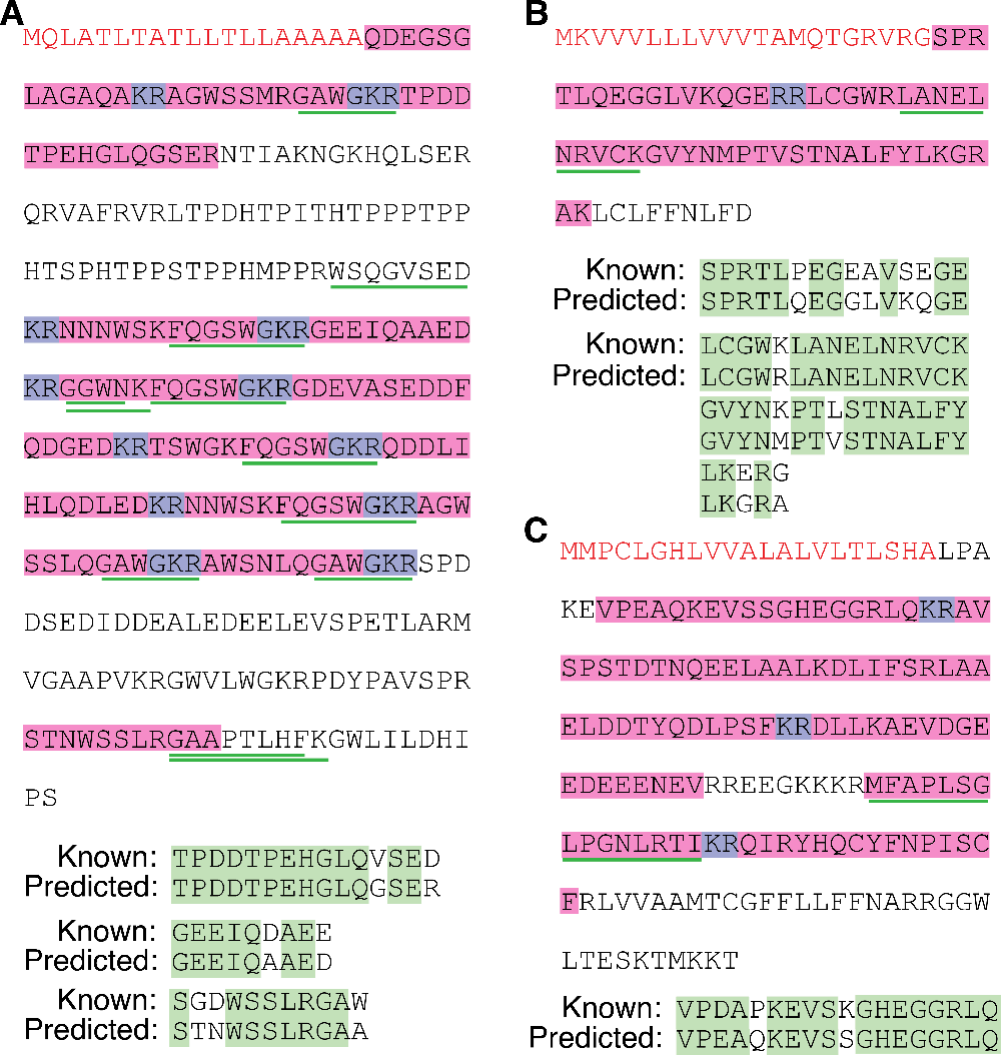
Peptides observed via mass spectrometry were used to map and extract mature neuropeptides from **A)** a natalisin precursor in *C. borealis*, **B)** an insulin-like peptide (ILP) in *C. sapidus*, and **C)** an allatostatin-C peptide in *C. sapidus*. Signal peptides are shown in red, predicted cleavage sites are highlighted in purple, and putative peptides are highlighted in pink. Green underlines indicate peptides detected by MS/MS database searching, exhibiting a 1 Da mass shift consistent with C-terminal amidation. The adjacent GKR motif denotes the predicted amidation and dibasic cleavage site. Green highlighting between known and predicted sequences indicates homology between experimentally discovered (‘known’) and *in silico* predicted (‘predicted’) peptides.

Similarly, the conserved GAW motif appeared within the same precursor, accompanied by a KR cleavage site approximately 6–7 residues upstream (Figure 3A). This organization produced peptides AGWSS**MR**GAWamide,^31,35^ AWS**NLQ**GAWamide, and AGWSS**LW**GAWamide, each flanked by KR and GKR at the N- and C-termini, respectively. Peptides AWS**NLQ**GAWamide and AGWSS**MR**GAWamide have previously been identified,^31,35^ as have the closely related isoforms AGWSS**LQ**GAWamide,^38,39^ AGWSS**LK**GAWamide,^31,35^ and AGWSS**TS**GAWamide.^40^ However, this is the first report of AGWSS**LW**GAWamide, a plausible isoform within the AST-B family. Also included within this precursor were peptides TPDDTPEHGLQ**GSER**, GEEIQ**AAED**, and S**TN**WSSLRGA**A** (Figure 3A), which are isoforms of known neuropeptides TPDDTPEHGLQ**VSED**,^35^ GEEIQ**DAEE**,^35^ and S**GD**WSSLRGA**W**,^15,31,32^ respectively. Curiously, TPDDTPEHGLQGSER, GEEIQAAED, and STNWSSLRGAA belong to the AST-B family, while the remaining peptides encoded on this precursor, including those bearing the QGSW and GAW motifs, correspond to natalisin neuropeptides. A similar event was also found in an RYamide putative precursor within *C. sapidus*, where two RFamide peptides and one RYamide peptide were encoded on a single precursor (Table S4).

We identified a similar trend in isoforms in a corazonin precursor within *C. sapidus* (Figure 3B). The precursor, obtained via homology with *Daphnia galeata*, documented as a pro-corazonin peptide (UniProt: A0A8J2RDF5), yielded a single peptide from the thoracic ganglion (TG), LANELNRVCK. While not immediately apparent, when mapped in context of its signal peptide on the precursor, two consecutive arginine residues were found upstream, producing peptides SPRTLQEGGLVKQGE and LCGWR**LANELNRVCK**GVYNMPTVSTNALFYLKGRA, each an isoform of peptides previously predicted via the *C. borealis* transcriptome.^37^ The latter peptide resembles the B-chain of an ILP, while the former is an ILP precursor-related peptide isoform. It is anticipated that the B-chain directly engages with an A-chain ILP, with the documented A-chain appearing as GLSAECCRKACSVSELAGYCY in the literature.^37^ Critically, the cysteines within the observed isoform are retained, suggesting preserved functionality.^37^ While the A chain was not observed, it can be hypothesized that further mechanistic studies may shed light on a comodulating peptide counterpart. Finally, another set of isoforms was established through an AST-C precursor, where several conserved, yet novel neuropeptides were observed in *C. sapidus* (Figure 3C). Altogether, this work provides a new space for neuropeptide discovery and for drawing functional connections.

### Motif Evaluation of Putative Peptides

Motifs are a central component of all neuropeptide research efforts. They have been used to identify peptides,^38^ hypothesize peptide function,^37^ and even bridge findings between vertebrates and invertebrates.^6^ Thus, we employed MotifQuest^41^ to search for prominent motifs within our predicted neuropeptides from *C. sapidus* and *C. borealis.*

In examining the MotifQuest results, we observed several notable motifs that are consistent with those previously reported. For example, the motif CYFNPISCF was found multiple times in the *C. sapidus* results, consistent with AST-C neuropeptides.^42^ Additionally, in *C. sapidus,* we identified known motifs such as FGXRL (*e.g.,* pyrokinin),^43^ YEXD and YDDD (*e.g.,* CHH amide),^41^ GASR, PSRA, and QGLG (*e.g.,* CPRP). In *C. borealis*, known motifs included those for tachykinin peptides: FLGMR, FYGXR, LGXR.^18^ CHHamide motifs were also identified, including WPPS, YEED, and GSLP. RYamide motifs were also identified, such as FYSQRY and GGXR.^44^ This level of similarity between predicted motifs and experimentally validated motifs was reflected in results obtained from a multiple sequence alignment between predicted and known motifs, showing a wide distribution of similarity scores centered around 50% for both species (Figure 4A). Upon closer examination, we observed the motif GSDES, which appeared 108 times within the *C. sapidus* predictions. While we focused mainly on full motifs or consecutive conserved regions of residues, partial motifs or a variable amino acid flanked by two conserved regions have been shown to convey peptide conservation.^41^ This same trend was evident in WebLogo analysis of all sequences, where the GSDES motif extended to a variable motif GXXGSDES, along with several other conserved regions (Figure 4B). In addition to GSDES, several other motifs were found with high frequency in *C. borealis* (Figure 4C) and *C. sapidus* (Figure 4D).

**Figure 4:**
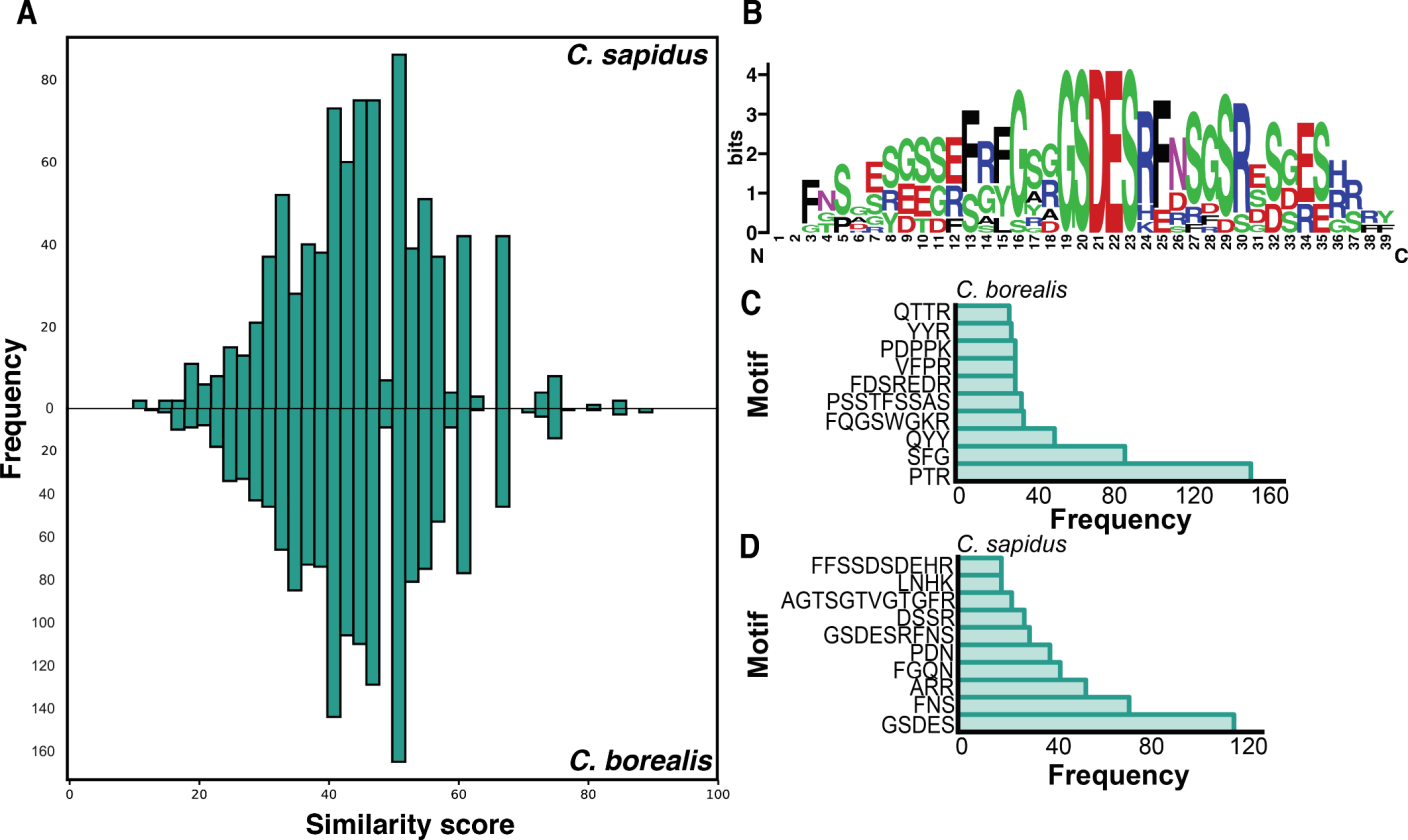
Analysis of conserved motifs in putative predicted neuropeptides. **A)** Fuzzy multiple sequence alignment between known neuropeptide motif database and predicted neuropeptides for *C. sapidus* (upper) and *C. borealis* (lower). **B)** WebLogo analysis of all matching peptides for motif GSDES, identified 108 times in *C. sapidus* predictions. Frequency of top ten motifs found for **C)** *C. borealis* and **D)** *C. sapidus.*.

### Global Perspective on Neuropeptide Identifications and Potential Functional Roles in Feeding

We observed a substantial increase in the number of identifications in the TG compared to the brain and SGs (Figure 5A), contrary to typical observations.^45^ Additionally, it was compelling that many peptides were detected across all tissues in both *C. borealis* (Table S5) and *C. sapidus* (Table S6). Given that feeding is a well-established context for crustacean neuropeptide signaling, we next evaluated our novel peptides in the context of feeding. As a pilot experiment to demonstrate the utility of our predicted peptide database in a biologically relevant context, we compared crabs dissected 30 min postfeeding with unfed controls and observed peptides with differential abundance across the TG, PO, and CoG tissues (Figure 5B). Three peptides HSNSRGSERamide (adipokinetic), QRLQWLR (AST-B), and RSSRQamide (RFamide-related) exhibited reduced abundances in the PO and elevated levels in the TG, while Q­(Gln→pyro-Glu)­VFEDRamide exhibited the opposite trend (*i.e.,* increased abundance in the PO and reduced levels in the TG), as determined by mean TIC-normalized intensity across technical replicates.

**Figure 5:**
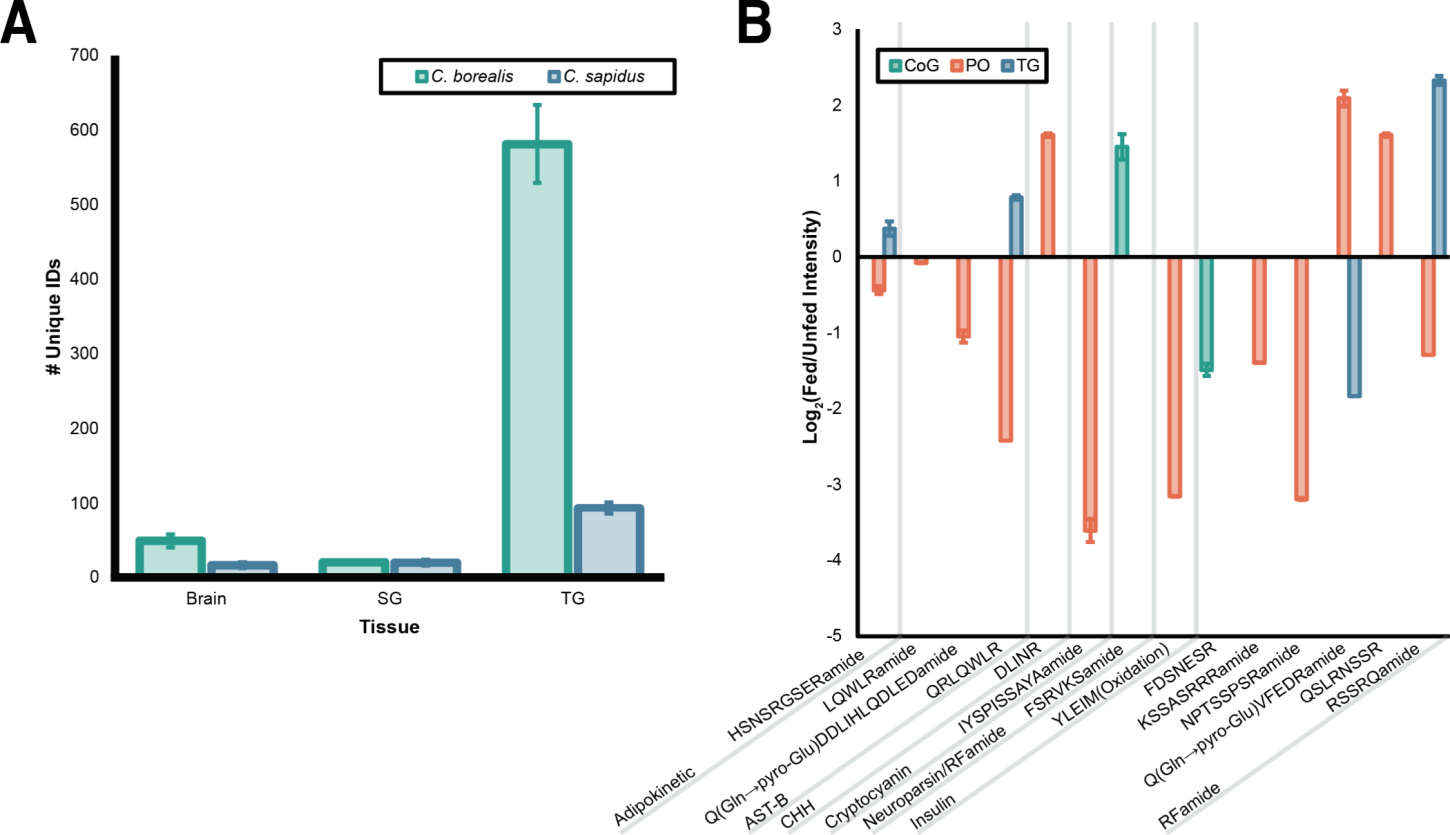
Biological analysis of predicted neuropeptides. **A)** Number of tissue-specific peptide identifications from the brain, sinus glands (SG), and thoracic ganglion (TG) in *C. borealis* and *C. sapidus*. **B)** Ratio of fed to unfed peptide abundance in *C. borealis* in commissural ganglion (CoG), pericardial organs (PO), and TG. Peptide families are noted below the corresponding peptides. Abbreviation: allatostatin-B (AST-B), crustacean hyperglycemic hormone (CHH). Error bars represent the standard deviation across technical replicates (*n* = 3).

Given the early stage of genome-enabled neuropeptide research in these species, we anticipated identifying neuropeptide isoforms, putative novel neuropeptides, and other biologically relevant variants made accessible by the recent release of both genomes. However, it should be noted that both genomes remain sparsely annotated, which limits the overall yield of predicted neuropeptides. Nevertheless, our study uncovered several novel candidate neuropeptides.

It is well-established that neuropeptide precursors can encode multiple neuropeptides; however, the extent and organization of this phenomenon are not well-understood. To address this, we mapped our predicted peptides onto their corresponding precursors. Natalisin peptides contain the conserved FXXXRamide motif at their C-termini, often occurring as multiple repeats within the same gene. Their amidation arises from a glycine followed by arginine and/or lysine at dibasic cleavage sites.^29^ Initially identified in insects, natalisin peptides have since been reported in crayfish through next-generation sequencing,^46^ and more recently through MS in the American lobster.^28^ To our knowledge, this is the first instance of natalisin peptide detection in *C. borealis*. This finding also provided new insight into precursor architecture, as we observed natalisin and AST-B peptides simultaneously encoded on the same neuropeptide precursor. This discovery is particularly intriguing, as genes are typically considered to encode a single neuropeptide family.^47,48^ To our knowledge, this represents one of the first documented cases in which two distinct peptide families are encoded on a single precursor, suggesting that coordination of distinct neuropeptide families within a single precursor may represent an underappreciated mechanism for neuropeptide signaling.

From a global perspective, we examined identifications obtained through database searches for putative neuropeptides in *C. borealis* and *C. sapidus* using their respective predicted databases. Database searching confirmed the presence of numerous predicted peptides in the empirical MS data sets from both species. Although there are relatively few reports on neuropeptides in the TG and the tissue is lipid rich, this framework suggests that this tissue may simply be unexplored, and that an unbiased genome-based search can provide deeper insight into its peptide composition.

In the context of feeding, four peptides showed differential abundance patterns between fed and unfed states, all observed in the PO and TG. It is well established that the PO is connected to the STNS, while the TG is connected to the brain.^49,50^ Thus, the observation that these two tissues, both peripheral to the heart, display opposing fold changes for the same peptides, is consistent with tissue-specific neuroendocrine roles for these peptides in the context of feeding. Mechanistic resolution of these patterns, for example, distinguishing increased secretion from reduced biosynthesis, will require transcript-level measurements such as RT-qPCR on prohormone mRNAs, which we identify as a priority for future studies.

In this work, we present the first, genome-derived prediction and experimental validation of the neuropeptidomes of *Callinectes sapidus* and *Cancer borealis*. By integrating a curated, multispecies neuropeptide precursor database with genomic alignment, signal peptide detection, and subsequent cleavage prediction, we generated a database of putative neuropeptides from two newly assembled and sparsely annotated crustacean genomes. This approach yielded more than 23,000 predicted peptides across both species, including numerous mature neuropeptide sequences and several previously unreported peptide families and isoforms. Collectively, these findings expand the known neuropeptide landscape in crustaceans, illuminating the rich peptide diversity encoded within newly sequenced genomes, and demonstrating the power of combining *in silico* genomic prediction with experimental MS. This work provides a valuable resource for future studies on neuropeptide evolution, signaling, and physiology, opening new avenues for mechanistic exploration of peptide function in the STNS, and related neuroendocrine organs, establishing a framework readily applicable to other newly sequenced invertebrate species.

## Methods

### Ethical Use of Animals in Research

No institutional approval is required for working with invertebrates, and all experiments were performed under national and local guidelines and regulations.

### Putative precursor identification

For alignment of the genomes with known neuropeptides, tBLASTn (National Center for Biotechnology Information, Bethesda, MD; http://blast.ncbi.nlm.nih.gov/Blast.cgi) was used, restricting to data from either *Callinectes sapidus* (GCA_020233015.1) or *Cancer borealis* (GCA_041682235.1) species. Query neuropeptide precursor sequences corresponding to crustacean sequences were extracted via UniProt. Query precursors were obtained via three search strategies: searching for the term “neuropeptide” in UniProt, filtering for crustacean species; searching for particular neuropeptide families in UniProt by name as a keyword, filtering for crustacean species; and referencing the recent publication of the lobster neuropeptidome.^28^ The neuropeptide families queried were the following: adipokinetic, allatostatin, bursicon, crustacean cardioactive peptide (CCAP), crustacean hyperglycemic hormone (CHH), corazonin, CHH precursor-related peptide (CPRP), diuretic hormone-31 (DH-31), ecdysis, elevin, FLRFamide, FMRFamide, gonadoliberin, GSEFLamide, HIGSLYRamide, insulin, molt-inhibiting hormone (MIH), mandibular organ-inhibiting hormone (MOIH), myosuppressin, natalisin, neuroparsin, orcokinin, orcomyotropin, proctolin, pyrokinin, RFamide, red pigment concentrating hormone (RPCH), RYamide, SIFamide, short neuropeptide F (sNPF), sulfakinin, tachykinin, and trissin.

Aligned sequences were filtered to an E-value less than 6 for loose searches, and less than 0.001 for restricted searches. Alignment was conducted using both PAM30 and BLOSUM62 algorithms, accommodating neuropeptide precursors of a variety of lengths.^51^ Sequence identity was also filtered to exclude hits with sequence identity less than 50%. Subsequently, aligned portions were translated and assessed for signal peptides using SignalP 6.0.^52^ Proteins containing a signal peptide with a confidence score of 0.70 or greater were retained. Remaining candidate protein sequences were cleaved using NeuroPred^27^ and evaluated for sequence motifs.

### Novel Peptide Motif Evaluation

MotifQuest was used to evaluate potential novel motifs that may be present in these identifications. In brief, peptide sequences were parsed from predicted neuropeptide databases using the SeqIO.parse function in BioPython,^53^ after which all sequences were aligned with Clustal Omega.^54^ Following alignment, pairwise evolutionary divergence between sequences was quantified using the ClustalW distance matrix function,^55^ which reports amino acid positional differences, where lower values indicate greater similarity. ClustalW was selected for integration into the MotifQuest workflow because it measures sequence similarity within an evolutionary context, consistent with the premise that conserved motifs arise from evolutionary pressures.^54^ Hierarchical clustering was then performed using the SciPy fcluster function^56^ applied to the ClustalW-generated distance matrix, with the clustering threshold (T-value) determining how the dendrogram was segmented into distinct motif groups.^56^ This approach allowed identification of related sequence families, paralleling the method used to classify neuropeptides.^57^ MotifQuest scored the resulting motifs using a framework that extends the weighted coverage method previously reported^38^ by incorporating a normalization step based on motif frequency within the full database. Motifs were ranked according to frequency of occurrence within the predicted peptide database.

Similarity between predicted and known motifs was assessed using a fuzzy sequence similarity approach, obtained via the rapidfuzz Python package (v. 3.14.1), which assigned a similarity score of 0–100 based on weighted positional identity, accommodating partial matches at variable positions. For WebLogo analysis, sequences extending 20 residues upstream and downstream of the cleavage site were manually extracted and aligned, setting the cleavage position to zero.

### Identification of Putative Mature Neuropeptides

Putative mature neuropeptides were identified based on several criteria: (i) cleavage at mono- or dibasic residues located at the N- and C-termini; (ii) for peptide families known to undergo C-terminal amidation (*e.g.,* RFamide, RYamide, AST-B), the presence of a C-terminal +1 glycine followed by dibasic residues, consistent with established neuropeptide processing mechanisms,^58^ was required; (iii) aligned with sequence motifs reported for several crustacean neuropeptide families;^5,6^ and (iv) cross-referenced with published mature neuropeptides from other invertebrate species.^59^ For the purposes of classification, “peptide” refers to a fully processed, mature sequence. Sequences bearing canonical neuropeptide features but lacking complete processing evidence were classified as “peptide-like”. Partial MS/MS-identified sequences were labeled “peptide fragment”, and sequences derived from precursor flanking regions were categorized as “precursor-related peptide”.

### Sample Preparation

*C. sapidus* and *C. borealis* crabs were obtained from Global Market (Madison, WI) and housed in an artificial seawater tank at a salinity concentration of 30 ppt. Crabs were equilibrated for at least 2 weeks prior to sacrifice. Crabs were dissected following anesthetization on ice for 30 min. From both species, the brain, commissural ganglion (CoG), paired pericardial organs (PO), thoracic ganglion (TG), and paired sinus glands (SG) were obtained and heat stabilized via Denator.

Neuropeptides were extracted from tissue by probe sonication in acidified methanol followed by centrifugation for 1 h at 16k *×g*, 4 °C, and the supernatant was retained and dried. For each condition, tissues from three individual crabs were pooled to generate a single biological replicate. Following pooling, samples were desalted using OMIX C18 tips (Agilent) according to manufacturer instructions, eluting sequentially in 25%, 50%, and 75% acetonitrile in 0.1% formic acid in water. Prior to MS analysis, samples were reconstituted in 20 μL of 0.1% FA. To ensure consistency in quantity of peptides injected, samples were evaluated via nanodrop (NanoDrop One, Thermo Fisher Scientific) at 205 nm, reflecting peptide bond absorbance.

### Mass Spectrometry Data Acquisition

Untargeted neuropeptide profiling was performed using LC-MS/MS on a Thermo Q-Exactive HF mass spectrometer interfaced with a Dionex Ultimate 3000 liquid chromatography system. Peptide separation employed mobile phase A consisting of 0.1% FA in water and mobile phase B containing 0.1% FA in acetonitrile. The gradient program increased from 10% to 20% B over 70 min, followed by 20% to 95% B over an additional 20 min, at a constant flow rate of 300 nL/min. Full MS data were collected in profile mode from *m*/*z* 200 to *m*/*z* 2000 at a resolving power of 60,000. The automatic gain control (AGC) target for MS^1^ scans was set at 1 × 10^6^ with a maximum injection time of 250 ms. MS/MS spectra were acquired in centroid mode, selecting the ten most intense precursor ions for HCD fragmentation with a 30-s dynamic exclusion. For DDA, instrument parameters included a resolution of 15,000, a 2.0 Th isolation window, an NCE of 30, a maximum injection time of 120 ms, an AGC target of 2 × 10^5^, and a fixed first mass of *m*/*z* 100. All samples were analyzed in technical triplicate.

### Feeding Study

Prior to feeding experiments, Jonah crabs (*C. borealis*) were equilibrated and fed as described previously.^60^ An unfed control crab was also placed on ice at the same time. *C. borealis* crabs were fed 4 g thawed tilapia, allowed to rest for 30 min following completion of feeding, and subsequently sacrificed with the aforementioned tissue collection strategy. Tissues were stored at −80 °C until MS analysis. Crabs were dissected and tissues were prepared for MS analysis, as described herein. MS analysis was performed on a Thermo Q-Exactive mass spectrometer coupled to a Waters Acquity liquid chromatography system using the same parameters as those described within.

### Database Searching from Mass Spectrometry Data

EndoGenius was utilized to evaluate the presence of the predicted peptides within empirical data sets.^38^ Analysis parameters were described elsewhere.^41^ In brief, precursor and fragment ion tolerances were set to 20 ppm and 0.02 Da, respectively. Variable PTMs included C-terminal amidation, oxidation of M, pyro-Glu from E, and pyro-Glu from Q. All identifications were obtained at an EndoGenius score threshold of 1000.

### Quantification and Statistics

For peptide quantification, abundances were normalized against total ion current (TIC) prior to evaluation. Peptide abundances were quantified using the precursor intensity extracted from the.MS2 format spectral files by EndoGenius. Precursor intensities were subsequently normalized to the TIC of each technical replicate to account for run-to-run variation. Prior to MS analysis, sample concentrations were normalized by nanodrop to ensure equivalent injection amounts across technical replicates.

To quantify, the peptides were required to be identified in at least two of the three technical replicates. Abundance was determined as the mean TIC normalized precursor intensity ± the standard deviation. For feeding calculations, the ratio of fed/unfed abundance was calculated as the log_2_ of the mean TIC-normalized intensity in the fed condition divided by that in the unfed condition. To avoid undefined values for zero-intensity observations, a small pseudo count offset (10^–8^) was applied. Error bars were calculated as the propagated standard error of the mean across technical replicates.

## Supplementary Material









## Data Availability

All mass spectrometry proteomics data have been deposited to the ProteomeXchange Consortium via the MassIVE partner repository with the data set identifier: MSV000100298. All code for data analysis and figure generation is available via GitHub at https://github.com/lingjunli-research/genome-guided-crustacean-neuropeptide-prediction.

## Notes

Multispecies database generation (Figure S1), predicted neuropeptide comparison (Figure S2), precursor sequence evaluation (Figure S3), mature neuropeptides (Tables S1 and S2), annotated neuropeptide precursors (Tables S3 and S4), and commonly found precursors (Tables S5 and S6) (PDF)
Multispecies database searched (Supplemental File 1) (XLSX)
C. borealis neuropeptide predictions (Supplemental File 2) (XLSX)
C. sapidus neuropeptide predictions (Supplemental File 3) (XLSX)
