# Supplementary material for: Neuropeptide Diversity Encoded in Newly Sequenced Crustacean Genomes Reveals Signaling Roles during Feeding
Source: ACS Chem Neurosci. 2026 Jun 11;17(13):2494–503. doi: 10.1021/acschemneuro.6c00123 (PMC13329902; doi:10.1021/acschemneuro.6c00123)
Supplement: Supplementary file 1 [file cn6c00123_si_001.pdf]

Supporting Information

Neuropeptide diversity encoded in newly sequenced crustacean genomes reveals signaling roles during feeding

Lauren Fields,[1] Vu Ngoc Huong Tran,[2] Thao Duong,[1] Tina C. Dang,[2] Kendra G. Selby,[1] Lingjun Li[1,2,3,4*]

[1]Department of Chemistry, 1101 University Avenue, University of Wisconsin, Madison, WI, 53706, USA.

[2]School of Pharmacy, 777 Highland Avenue, University of Wisconsin, Madison, WI, 53705, USA.

[3]Lachman Institute for Pharmaceutical Development, School of Pharmacy, University of Wisconsin-Madison, Madison, WI 53705, USA.

[4]Wisconsin Center for NanoBioSystems, School of Pharmacy, University of Wisconsin-Madison, Madison, WI 53705, USA.

[*]Corresponding author

Tel.: +1 (608) 265-8491

Fax: +1 (608) 262-5345

E-mail: lingun.li@wisc.edu

Table of Contents

Supplemental Figures (located within this document)

- **Figure S1:** Crustacean species used for broad database generation.
- **Figure S2:** Comparison of predicted neuropeptides in *C. borealis and C. sapidus*.
- **Figure S3:** Peptide-flanking precursor sequences in *C. borealis and C. sapidus.*
- **Table S1:** Mature neuropeptides obtained from *C. borealis*.
- **Table S2:** Mature neuropeptides obtained from *C. sapidus*.
- **Table S3:** Annotated *C. borealis* neuropeptide precursors.
- **Table S4:** Annotated *C. sapidus* neuropeptide precursors.
- **Table S5:** Commonly found precursors in *C. borealis* tissues, with their peptides.
- **Table S6:** Commonly found precursors in *C. sapidus* tissues, with their peptides.

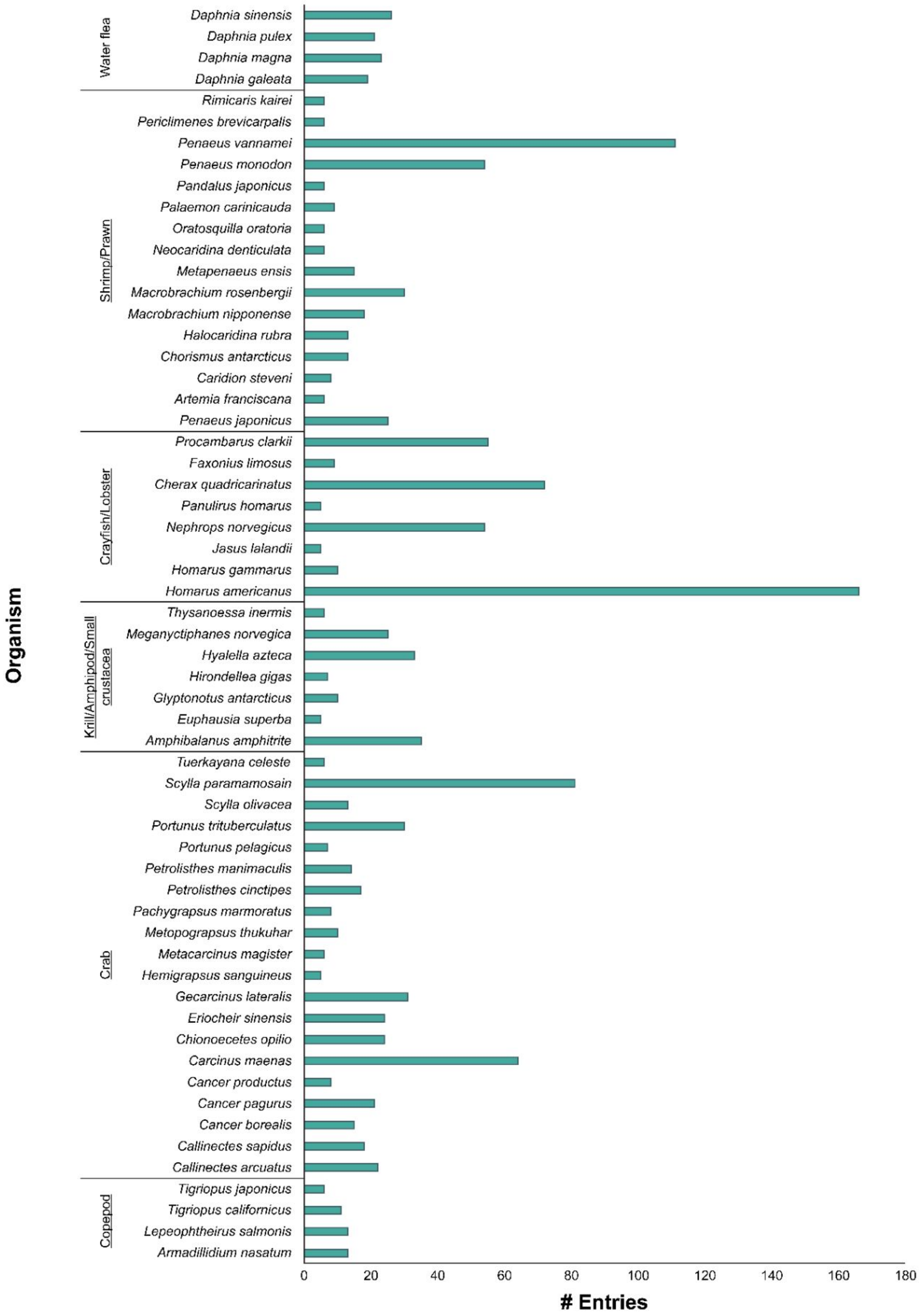

Figure S1: Organisms contributing to the alignment.

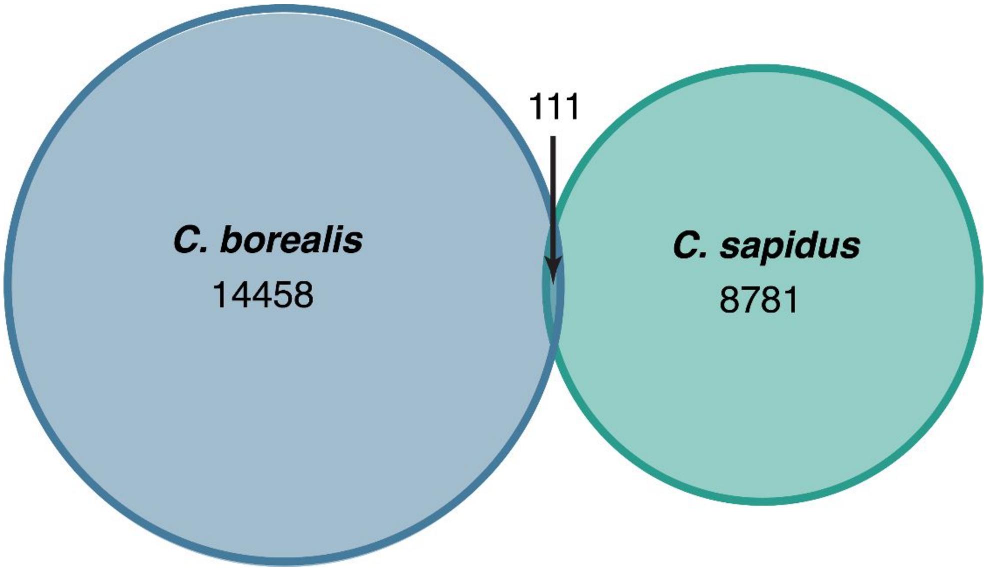

Figure S2: Overlap of putative neuropeptides between *C. borealis* and *C. sapidus*.

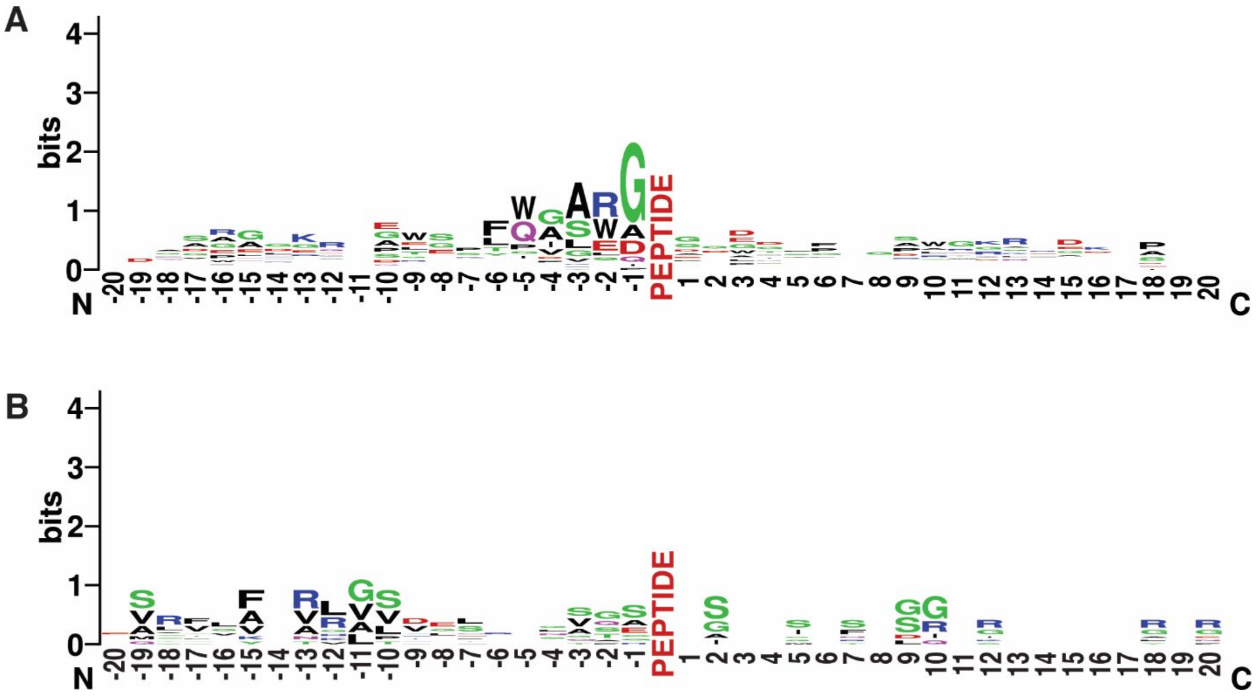

Figure S3: Alignment surrounding peptides within the precursor of **A)** *C. borealis* and **B)** *C. sapidus*.

Sequence	Family	Precursor	SP Length	Gene ID	Scaffold	Chromosome
QEVSPG<br>EAGGSE<br>GHSGAA<br>APWVGQ<br>RHA	Natalisin	MDSVWLLLVMAL<br>AATLAATAQEVSP<br>GEAGGSEGHSGA<br>AAPWVGQRHARS<br>LEAGGDASWLPV<br>VQDEEVTQPVSG<br>KGAGHGGTTFWV<br>ARGKKDAEGSLS<br>YYWGPNQSLWG<br>GEASHRGGPSMS<br>NSPYVPLLQKSG<br>WESNPSLWGKRD<br>GHGPFWAARGKR<br>PERDPFWVSRGR<br>RDLAEAPPAPLHQ<br>WVAEDPAAQEHK<br>DEQLWGGEIKRE<br>EGGPFWISRGKR<br>PQPGSAASQLASL<br>WAVRGRKSGADN<br>TFWVARGKKETD<br>PRGPFWAARGRR<br>SGGEGGTGPYWI<br>ARGKKQDGTTPT<br>GPYWIARGKKGD<br>EDSVFWAARGKK<br>DPPAWPTGRGRR<br>EDETHSFWIARGK<br>KSGAITPRTAEKD<br>NDDQGDNDEQHE	20-21	g28710	15	23

Sequence	Family	Precursor	SP Length	Gene ID	Scaffold	Chromosome
		EAMQKATDNYLK GFTTLAE				
SLEAGG DASWLP VVQDEE VTQPVS G	Natalisin	MDSVWLLLVMAL AATLAATAQEVSP GEAGGSEGHSGA AAPWVGQRHARS LEAGGDASWLPV VQDEEVTQPVSG KGAGHGGTTFWV ARGKKDAEGSLS YYWGPNQSLWG GEASHRGGPSMS NSPYVPLLQKSG WESNPSLWGKRD GHGPFWAARGKR PERDPFWVSRGR RDLAEAPPAPLHQ WVAEDPAAQEHK DEQLWGGEIKRE EGGPFWISRGKR PQPGSAASQLASL WAVRGRKSGADN TFWVARGKKETD PRGPFWAARGRR SGGEGGTGPYWI ARGKKQDGTTPT GPYWIARGKKGD EDSVFWAARGKK DPPAWPTGRGRR EDETHSFWIARGK KSGAITPRTAEKD	20-21	g28710	15	23

Sequence	Family	Precursor	SP Length	Gene ID	Scaffold	Chromosome
		NDDQGDNDEQHE EAMQKATDNYLK GFTTLAE				
GAGHGG TTFWVA R	Natalisin	MDSVWLLLVMAL AATLAATAQEVSP GEAGGSEGHSGA AAPWVGQRHARS LEAGGDASWLPV VQDEEVTQPVSG KGAGHGGTTFWV ARGKKDAEGSLS YYWGPNQSLWG GEASHRGGPSMS NSPYVPLLQKSG WESNPSLWGKRD GHGPFWAARGKR PERDPFWVSRGR RDLAEAPPAPLHQ WVAEDPAAQEHK DEQLWGGEIKRE EGGPFWISRGKR PQPGSAASQLASL WAVRGRKSGADN TFWVARGKKETD PRGPFWAARGRR SGGEGGTGPYWI ARGKKQDGTTPT GPYWIARGKKGD EDSVFWAARGKK DPPAWPTGRGRR EDETHSFWIARGK	20-21	g28710	15	23

Sequence	Family	Precursor	SP Length	Gene ID	Scaffold	Chromosome
		KSGAITPRTAEKD NDDQGDNDEQHE EAMQKATDNYLK GFTTLAE				
SGWESN PSLW	AST-B	MDSVWLLLVMAL AATLAATAQEVSP GEAGGSEGHSGA AAPWVGQRHARS LEAGGDASWLPV VQDEEVTQPVSG KGAGHGGTTFWV ARGKKDAEGSLS YYWGPNQSLWG GEASHRGGPSMS NSPYVPLLQKSG WESNPSLWGKRD GHGPFWAARGKR PERDPFWVSRGR RDLAEAPPAPLHQ WVAEDPAAQEHK DEQLWGGEIKRE EGGPFWISRGKR PQPGSAASQLASL WAVRGRKSGADN TFWVARGKKETD PRGPFWAARGRR SGGEGGTGPYWI ARGKKQDGTTPT GPYWIARGKKGD EDSVFWAARGKK DPPAWPTGRGRR	20-21	g28710	15	23

Sequence	Family	Precursor	SP Length	Gene ID	Scaffold	Chromosome
		EDETHSFWIARGK KSGAITPRTAEKD NDDQGDNDEQHE EAMQKATDNYLK GFTTLAE				
DGHGPF WAAR	Natalisin	MDSVWLLLVMAL AATLAATAQEVSP GEAGGSEGHSGA AAPWVGQRHARS LEAGGDASWLPV VQDEEVTQPVSG KGAGHGGTTFWV ARGKKDAEGSLS YYWGPNQSLWG GEASHRGGPSMS NSPYVPLLQKSG WESNPSLWGKRD GHGPFWAARGKR PERDPFWVSRGR RDLAEAPPAPLHQ WVAEDPAAQEHK DEQLWGGEIKRE EGGPFWISRGKR PQPGSAASQLASL WAVRGRKSGADN TFWVARGKKETD PRGPFWAARGRR SGGEGGTGPYWI ARGKKQDGTTPT GPYWIARGKKGD EDSVFWAARGKK	20-21	g28710	15	23

Sequence	Family	Precursor	SP Length	Gene ID	Scaffold	Chromosome
		DPPAWPTGRGRR EDETHSFWIARGK KSGAITPRTAEKD NDDQGDNDEQHE EAMQKATDNYLK GFTTLAE				
EEGGPF WISR	Natalisin	MDSVWLLLVMAL AATLAATAQEVSP GEAGGSEGHSGA AAPWVGQRHARS LEAGGDASWLPV VQDEEVTQPVSG KGAGHGGTTFWV ARGKKDAEGSLS YYWGPNQSLWG GEASHRGGPSMS NSPYVPLLQKSG WESNPSLWGKRD GHGPFWAARGKR PERDPFWVSRGR RDLAEAPPAPLHQ WVAEDPAAQEHK DEQLWGGEIKRE EGGPFWISRGKR PQPGSAASQLASL WAVRGRKSGADN TFWVARGKKETD PRGPFWAARGRR SGGEGGTGPYWI ARGKKQDGTTPT GPYWIARGKKGD	20-21	g28710	15	23

Sequence	Family	Precursor	SP Length	Gene ID	Scaffold	Chromosome
		EDSVFWAARGKK DPPAWPTGRGRR EDETHSFWIARGK KSGAITPRTAEKD NDDQGDNDEQHE EAMQKATDNYLK GFTTLAE				
PQPGSA ASQLASL WAVR	Natalisin	MDSVWLLLVMAL AATLAATAQEVSP GEAGGSEGHSGA AAPWVGQRHARS LEAGGDASWLPV VQDEEVTQPVSG KGAGHGGTTFWV ARGKKDAEGSLS YYWGPNQSLWG GEASHRGGPSMS NSPYVPLLQKSG WESNPSLWGKRD GHGPFWAARGKR PERDPFWVSRGR RDLAEAPPAPLHQ WVAEDPAAQEHK DEQLWGGEIKRE EGGPFWISRGKR PQPGSAASQLASL WAVRGRKSGADN TFWVARGKKETD PRGPFWAARGRR SGGEGGTGPYWI ARGKKQDGTTPT	20-21	g28710	15	23

Sequence	Family	Precursor	SP Length	Gene ID	Scaffold	Chromosome
		GPYWIARGKKGD EDSVFWAARGKK DPPAWPTGRGRR EDETHSFWIARGK KSGAITPRTAEKD NDDQGDNDEQHE EAMQKATDNYLK GFTTLAE				
SGADNT FWVAR	Natalisin	MDSVWLLLVMAL AATLAATAQEVSP GEAGGSEGHSGA AAPWVGQRHARS LEAGGDASWLPV VQDEEVTQPVSG KGAGHGGTTFWV ARGKKDAEGSLS YYWGPNQSLWG GEASHRGGPSMS NSPYVPLLQKSG WESNPSLWGKRD GHGPFWAARGKR PERDPFWVSRGR RDLAEAPPAPLHQ WVAEDPAAQEHK DEQLWGGEIKRE EGGPFWISRGKR PQPGSAASQLASL WAVRGRKSGADN TFWVARGKKETD PRGPFWAARGRR SGGEGGTGPYWI	20-21	g28710	15	23

Sequence	Family	Precursor	SP Length	Gene ID	Scaffold	Chromosome
		ARGKKQDGTTPT GPYWIARGKKGD EDSVFWAARGKK DPPAWPTGRGRR EDETHSFWIARGK KSGAITPRTAEKD NDDQGDNDEQHE EAMQKATDNYLK GFTTLAE				
SGGEGG TGPYWIA R	Natalisin	MDSVWLLLVMAL AATLAATAQEVSP GEAGGSEGHSGA AAPWVGQRHARS LEAGGDASWLPV VQDEEVTQPVSG KGAGHGGTTFWV ARGKKDAEGSLS YYWGPNQSLWG GEASHRGGPSMS NSPYVPLLQKSG WESNPSLWGKRD GHGPFWAARGKR PERDPFWVSRGR RDLAEAPPAPLHQ WVAEDPAAQEHK DEQLWGGEIKRE EGGPFWISRGKR PQPGSAASQLASL WAVRGRKSGADN TFWVARGKKETD PRGPFWAARGRR	20-21	g28710	15	23

Sequence	Family	Precursor	SP Length	Gene ID	Scaffold	Chromosome
		SGGEGGTGPYWI ARGKKQDGTTPT GPYWIARGKKGD EDSVFWAARGKK DPPAWPTGRGRR EDETHSFWIARGK KSGAITPRTAEKD NDDQGDNDEQHE EAMQKATDNYLK GFTTLAE				
QDGTTP TGPYWIA R	Natalisin	MDSVWLLLVMAL AATLAATAQEVSP GEAGGSEGHSGA AAPWVGQRHARS LEAGGDASWLPV VQDEEVTQPVSG KGAGHGGTTFWV ARGKKDAEGSLS YYWGPNQSLWG GEASHRGGPSMS NSPYVPLLQKSG WESNPSLWGKRD GHGPFWAARGKR PERDPFWVSRGR RDLAEAPPAPLHQ WVAEDPAAQEHK DEQLWGGEIKRE EGGPFWISRGKR PQPGSAASQLASL WAVRGRKSGADN TFWVARGKKETD	20-21	g28710	15	23

Sequence	Family	Precursor	SP Length	Gene ID	Scaffold	Chromosome
		PRGPFWAARGRR SGGEGGTGPYWI ARGKKQDGTTPT GPYWIARGKKGD EDSVFWAARGKK DPPAWPTGRGRR EDETHSFWIARGK KSGAITPRTAEKD NDDQGDNDEQHE EAMQKATDNYLK GFTTLAE				
GDEDSV FWAAR	Natalisin	MDSVWLLLVMAL AATLAATAQEVSP GEAGGSEGHSGA AAPWVGQRHARS LEAGGDASWLPV VQDEEVTQPVSG KGAGHGGTTFWV ARGKKDAEGSLS YYWGPNQSLWG GEASHRGGPSMS NSPYVPLLQKSG WESNPSLWGKRD GHGPFWAARGKR PERDPFWVSRGR RDLAEAPPAPLHQ WVAEDPAAQEHK DEQLWGGEIKRE EGGPFWISRGKR PQPGSAASQLASL WAVRGRKSGADN	20-21	g28710	15	23

Sequence	Family	Precursor	SP Length	Gene ID	Scaffold	Chromosome
		TFWVARGKKETD<br>PRGPFWAARGRR<br>SGGEGGTGPYWI<br>ARGKKQDGTTPT<br>GPYWIARGKKGD<br>EDSVFWAARGKK<br>DPPAWPTGRGRR<br>EDETHSFWIARGK<br>KSGAITPRTAEKD<br>NDDQGDNDEQHE<br>EAMQKATDNYLK<br>GFTTLAE				
EDETHSF<br>WIAR	Natalisin	MDSVWLLLVMAL<br>AATLAATAQEVSP<br>GEAGGSEGHSGA<br>AAPWVGQRHARS<br>LEAGGDASWLPV<br>VQDEEVTQPVSG<br>KGAGHGGTTFWV<br>ARGKKDAEGSLS<br>YYWGPNQSLWG<br>GEASHRGGPSMS<br>NSPYVPLLQKSG<br>WESNPSLWGKRD<br>GHGPFWAARGKR<br>PERDPFWVSRGR<br>RDLAEAPPAPLHQ<br>WVAEDPAAQEHK<br>DEQLWGGEIKRE<br>EGGPFWISRGKR<br>PQPGSAASQLASL	20-21	g28710	15	23

Sequence	Family	Precursor	SP Length	Gene ID	Scaffold	Chromosome
		WAVRGRKSGADN TFWVARGKKETD PRGPFWAARGRR SGGEGGTGPYWI ARGKKQDGTTPT GPYWIARGKKGD EDSVFWAARGKK DPPAWPTGRGRR EDETHSFWIARGK KSGAITPRTAEKD NDDQGDNDEQHE EAMQKATDNYLK GFTTLAE				
PERDPF WVSR	Natalisin	MDSVWLLLVMAL AATLAATAQEVSP GEAGGSEGHSGA AAPWVGQRHARS LEAGGDASWLPV VQDEEVTQPVSG KGAGHGGTTFWV ARGKKDAEGSLS YYWGPNQSLWG GEASHRGGPSMS NSPYVPLLQKSG WESNPSLWGKRD GHGPFWAARGKR PERDPFWVSRGR RDLAEAPPAPLHQ WVAEDPAAQEHK DEQLWGGEIKRE EGGPFWISRGKR	20-21	g28710	15	23

Sequence	Family	Precursor	SP Length	Gene ID	Scaffold	Chromosome
		PQPGSAASQLASLWAVRGRKSGADNTFWVARGKKETDPRGPFWAARGRRSGGEGGTGPYWIARGKKQDGTTPTGPYWIARGKKGDEDSVFWAARGKKDPPAWPTGRGRREDETHSFWIARGKKSGAITPRTAEKDNDDQGDNDEQHEEAMQKATDNYLKGFTTLAE				
DLAEAPPAPLHQWVAEDPAAQEHKDEQLWGGEI	Natalisin	MDSVWLLLVMALAATLAATAQEVSPGEAGGSEGHSGAAAPWVGQRHARSLEAGGDASWLPVVQDEEVTQPVSGKGAGHGGTTFWVARGKKDAEGSLSYYWGPNQSLWGGEASHRGGPSMSNSPYVPLLQKSGWESNPSLWGKRDGHGPFWAARGKRPERDPFWVSRGRRDLAEAPPAPLHQWVAEDPAAQEHKDEQLWGGEIKRE	20-21	g28710	15	23

Sequence	Family	Precursor	SP Length	Gene ID	Scaffold	Chromosome
		EGGPFWISRGKR PQPGSAASQLASL WAVRGRKSGADN TFWVARGKKETD PRGPFWAARGRR SGGEGGTGPYWI ARGKKQDGTTPT GPYWIARGKKGD EDSVFWAARGKK DPPAWPTGRGRR EDETHSFWIARGK KSGAITPRTAEKD NDDQGDNDEQHE EAMQKATDNYLK GFTTLAE				
ETDPRG PFWAAR	Natalisin	MDSVWLLLVMAL AATLAATAQEVSP GEAGGSEGHSGA AAPWVGQRHARS LEAGGDASWLPV VQDEEVTQPVSG KGAGHGGTTFWV ARGKKDAEGSLS YYWGPNQSLWG GEASHRGGPSMS NSPYVPLLQKSG WESNPSLWGKRD GHGPFWAARGKR PERDPFWVSRGR RDLAEAPPAPLHQ WVAEDPAAQEHK	20-21	g28710	15	23

Sequence	Family	Precursor	SP Length	Gene ID	Scaffold	Chromosome
		DEQLWGGEIKRE EGGPFWISRGKR PQPGSAASQLASL WAVRGRKSGADN TFWVARGKKETD PRGPFWAARGRR SGGEGGTGPYWI ARGKKQDGTTPT GPYWIARGKKGD EDSVFWAARGKK DPPAWPTGRGRR EDETHSFWIARGK KSGAITPRTAEKD NDDQGDNDEQHE EAMQKATDNYLK GFTTLAE				
SGAITPR TAEKDN DDQGDN DEQHEE AMQ	Natalisin	MDSVWLLLVMAL AATLAATAQEVSP GEAGGSEGHSGA AAPWVGQRHARS LEAGGDASWLPV VQDEEVTQPVSG KGAGHGGTTFWV ARGKKDAEGSLS YYWGPNQSLWG GEASHRGGPSMS NSPYVPLLQKSG WESNPSLWGKRD GHGPFWAARGKR PERDPFWVSRGR RDLAEAPPAPLHQ	20-21	g28710	15	23

Sequence	Family	Precursor	SP Length	Gene ID	Scaffold	Chromosome
		WVAEDPAAQEHK DEQLWGGEIKRE EGGPFWISRGKR PQPGSAASQLASL WAVRGRKSGADN TFWVARGKKETD PRGPFWAARGRR SGGEGGTGPYWI ARGKKQDGTTPT GPYWIARGKKGD EDSVFWAARGKK DPPAWPTGRGRR EDETHSFWIARGK KSGAITPRTAEKD NDDQGDNDEQHE EAMQKATDNYLK GFTTLAE				
QDEGSG LAGAQA	AST-B	MQLATLTATLLTLL AAAAAQDEGSGL AGAQAKRAGWSS MRGAWGKRTPDD TPEHGLQGSERN TIAKNGKHQLSER QRVAFRVRLTPDH TPITHTPPPTPPHT SPHTPPSTPPHMP PRWSQGVSEDKR NNNWSKFQGSW GKRGEEIQAAEDK RGGWNKFQGSW GKRGDEVASEDD	19-20	g30501	28	24

Sequence	Family	Precursor	SP Length	Gene ID	Scaffold	Chromosome
		FQDGEDKRTSWG KFQGSWGKRQDD LIHLQDLEDKRNN WSKFQGSWGKR AGWSSLQGAWG KRAWSNLQGAW GKRSPDDSEDIDD EALEDEELEVSPE TLARMVGAAPVK RGWVLWGKRPDY PAVSPRSTNWSS LRGAAPTLHFKG WLILDHIPS*				
TPDDTPE HGLQGS E	AST-B	MQLATLTATLLTLL AAAAAQDEGSGL AGAQAKRAGWSS MRGAWGKRTPDD TPEHGLQGSERN TIAKNGKHQLSER QRVAFRVRLTPDH TPITHTPPPTPPHT SPHTPPSTPPHMP PRWSQGVSEDKR NNNWSKFQGSW GKRGEEIQAAEDK RGGWNKFQGSW GKRGDEVASEDD FQDGEDKRTSWG KFQGSWGKRQDD LIHLQDLEDKRNN WSKFQGSWGKR	19-20	g30501	28	24

Sequence	Family	Precursor	SP Length	Gene ID	Scaffold	Chromosome
		AGWSSLQGAWG KRAWSNLQGAW GKRSPDDSEDIDD EALEDEELEVSPE TLARMVGAAPVK RGWVLWGKRPDY PAVSPRSTNWSS LRGAAPTLHFKG WLILDHIPS				
GEEIQAA ED	AST-B	MQLATLTATLLTLL AAAAAQDEGSGL AGAQAKRAGWSS MRGAWGKRTPDD TPEHGLQGSERN TIAKNGKHQLSER QRVAFRVRLTPDH TPITHTPPPTPPHT SPHTPPSTPPHMP PRWSQGVSEDKR NNNWSKFQGSW GKRGEEIQAAEDK RGGWNKFQGSW GKRGDEVASEDD FQDGEDKRTSWG KFQGSWGKRQDD LIHLQDLEDKRNN WSKFQGSWGKR AGWSSLQGAWG KRAWSNLQGAW GKRSPDDSEDIDD EALEDEELEVSPE	19-20	g30501	28	24

Sequence	Family	Precursor	SP Length	Gene ID	Scaffold	Chromosome
		TLARMVGAAPVK RGWVLWGKRPDY PAVSPRSTNWSS LRGAAPTLHFKG WLILDHIPS*				
GDEVAS EDDFQD GED	AST-B	MQLATLTATLLTLL AAAAAQDEGSGL AGAQAKRAGWSS MRGAWGKRTPDD TPEHGLQGSERN TIAKNGKHQLSER QRVAFRVRLTPDH TPITHTPPPTPPHT SPHTPPSTPPHMP PRWSQGVSEDKR NNNWSKFQGSW GKRGEEIQAAEDK RGGWNKFQGSW GKRGDEVASEDD FQDGEDKRTSWG KFQGSWGKRQDD LIHLQDLEDKRNN WSKFQGSWGKR AGWSSLQGAWG KRAWSNLQGAW GKRSPDDSEDIDD EALEDEELEVSPE TLARMVGAAPVK RGWVLWGKRPDY PAVSPRSTNWSS	19-20	g30501	28	24

Sequence	Family	Precursor	SP Length	Gene ID	Scaffold	Chromosome
		LRGAAPTLHFKG WLILDHIPS				
QDDLIHL QDLED	AST-B	MQLATLTATLLTLL AAAAAQDEGSGL AGAQAKRAGWSS MRGAWGKRTPDD TPEHGLQGSERN TIAKNGKHQLSER QRVAFRVRLTPDH TPITHTPPPTPPHT SPHTPPSTPPHMP PRWSQGVSEDKR NNNWSKFQGSW GKRGEEIQAAEDK RGGWNKFQGSW GKRGDEVASEDD FQDGEDKRTSWG KFQGSWGKRQDD LIHLQDLEDKRNN WSKFQGSWGKR AGWSSLQGAWG KRAWSNLQGAW GKRSPDDSEDIDD EALEDEELEVSPE TLARMVGAAPVK RGWVLWGKRPDY PAVSPRSTNWSS LRGAAPTLHFKG WLILDHIPS*	19-20	g30501	28	24
AGWSSL QGAW	AST-B	MQLATLTATLLTLL AAAAAQDEGSGL	19-20	g30501	28	24

Sequence	Family	Precursor	SP Length	Gene ID	Scaffold	Chromosome
		AGAQAKRAGWSS MRGAWGKRTPDD TPEHGLQGSERN TIAKNGKHQLSER QRVAFRVRLTPDH TPITHTPPPTPPHT SPHTPPSTPPHMP PRWSQGVSEDKR NNNWSKFQGSW GKRGEEIQAAEDK RGGWNKFQGSW GKRGDEVASEDD FQDGEDKRTSWG KFQGSWGKRQDD LIHLQDLEDKRNN WSKFQGSWGKR AGWSSLQGAWG KRAWSNLQGAW GKRSPDDSEDIDD EALEDEELEVSPE TLARMVGAAPVK RGWVLWGKRPDY PAVSPRSTNWSS LRGAAPTLHFKG WLILDHIPS*				
AWSNLQ GAW	AST-B	MQLATLTATLLTLL AAAAAQDEGSGL AGAQAKRAGWSS MRGAWGKRTPDD TPEHGLQGSERN TIAKNGKHQLSER	19-20	g30501	28	24

Sequence	Family	Precursor	SP Length	Gene ID	Scaffold	Chromosome
		QRVAFRVRLTPDH<br>TPITHTPPPTPPHT<br>SPHTPPSTPPHMP<br>PRWSQGVSEDKR<br>NNNWSKFQGSW<br>GKRGEEIQAAEDK<br>RGGWNKFQGSW<br>GKRGDEVASEDD<br>FQDGEDKRTSWG<br>KFQGSWGKRQDD<br>LIHLQDLEDKRNN<br>WSKFQGSWGKR<br>AGWSSLQGAWG<br>KRAWSNLQGAW<br>GKRSPDDSEDIDD<br>EALEDEELEVSPE<br>TLARMVGAAPVK<br>RGWVLWGKRPDY<br>PAVSPRSTNWSS<br>LRGAAPTLHFKG<br>WLILDHIPS*				
SPDDSE<br>DIDDEAL<br>EDEELEV<br>SPETLA	AST-B	MQLATLTATLLTLL<br>AAAAAQDEGSGL<br>AGAQAKRAGWSS<br>MRGAWGKRTPDD<br>TPEHGLQGSERN<br>TIAKNGKHQLSER<br>QRVAFRVRLTPDH<br>TPITHTPPPTPPHT<br>SPHTPPSTPPHMP<br>PRWSQGVSEDKR	19-20	g30501	28	24

Sequence	Family	Precursor	SP Length	Gene ID	Scaffold	Chromosome
		NNNWSKFQGSW GKRGEEIQAAEDK RGGWNKFQGSW GKRGDEVASEDD FQDGEDKRTSWG KFQGSWGKRQDD LIHLQDLEDKRNN WSKFQGSWGKR AGWSSLQGAWG KRAWSNLQGAW GKRSPDDSEDIDD EALEDEELEVSPE TLARMVGAAPVK RGWVLWGKRPDY PAVSPRSTNWSS LRGAAPTLHFKG WLILDHIPS*				
AGWSSM RGAW	AST-B	MQLATLTATLLTLL AAAAAQDEGSGL AGAQAKRAGWSS MRGAWGKRTPDD TPEHGLQGSERN TIAKNGKHQLSER QRVAFRVRLTPDH TPITHTPPPTPPHT SPHTPPSTPPHMP PRWSQGVSEDKR NNNWSKFQGSW GKRGEEIQAAEDK RGGWNKFQGSW GKRGDEVASEDD	19-20	g30501	28	24

Sequence	Family	Precursor	SP Length	Gene ID	Scaffold	Chromosome
		FQDGEDKRTSWG KFQGSWGKRQDD LIHLQDLEDKRNN WSKFQGSWGKR AGWSSLQGAWG KRAWSNLQGAW GKRSPDDSEDIDD EALEDEELEVSPE TLARMVGAAPVK RGWVLWGKRPDY PAVSPRSTNWSS LRGAAPTLHFKG WLILDHIPS*				
TPDDTPE HGLQGS ERNTIAK N	AST-B	MQLATLTATLLTLL AAAAAQDEGSGL AGAQAKRAGWSS MRGAWGKRTPDD TPEHGLQGSERN TIAKNGKHQLSER QRVAFRVRLTPDH TPITHTPPPTPPHT SPHTPPSTPPHMP PRWSQGVSEDKR NNNWSKFQGSW GKRGEEIQAAEDK RGGWNKFQGSW GKRGDEVASEDD FQDGEDKRTSWG KFQGSWGKRQDD LIHLQDLEDKRNN WSKFQGSWGKR	19-20	g30501	28	24

Sequence	Family	Precursor	SP Length	Gene ID	Scaffold	Chromosome
		AGWSSLQGAWG KRAWSNLQGAW GKRSPDDSEDIDD EALEDEELEVSPE TLARMVGAAPVK RGWVLWGKRPDY PAVSPRSTNWSS LRGAAPTLHFKG WLILDHIPS*				
NNNWSK FQGSW	AST-B	MQLATLTATLLTLL AAAAAQDEGSGL AGAQAKRAGWSS MRGAWGKRTPDD TPEHGLQGSERN TIAKNGKHQLSER QRVAFRVRLTPDH TPITHTPPPTPPHT SPHTPPSTPPHMP PRWSQGVSEDKR NNNWSKFQGSW GKRGEEIQAAEDK RGGWNKFQGSW GKRGDEVASEDD FQDGEDKRTSWG KFQGSWGKRQDD LIHLQDLEDKRNN WSKFQGSWGKR AGWSSLQGAWG KRAWSNLQGAW GKRSPDDSEDIDD EALEDEELEVSPE	19-20	g30501	28	24

Sequence	Family	Precursor	SP Length	Gene ID	Scaffold	Chromosome
		TLARMVGAAPVK RGWVLWGKRPDY PAVSPRSTNWSS LRGAAPTLHFKG WLILDHIPS*				
GGWNKF QGSW	AST-B	MQLATLTATLLTLL AAAAAQDEGSGL AGAQAKRAGWSS MRGAWGKRTPDD TPEHGLQGSERN TIAKNGKHQLSER QRVAFRVRLTPDH TPITHTPPPTPPHT SPHTPPSTPPHMP PRWSQGVSEDKR NNNWSKFQGSW GKRGEEIQAAEDK RGGWNKFQGSW GKRGDEVASEDD FQDGEDKRTSWG KFQGSWGKRQDD LIHLQDLEDKRNN WSKFQGSWGKR AGWSSLQGAWG KRAWSNLQGAW GKRSPDDSEDIDD EALEDEELEVSPE TLARMVGAAPVK RGWVLWGKRPDY PAVSPRSTNWSS	19-20	g30501	28	24

Sequence	Family	Precursor	SP Length	Gene ID	Scaffold	Chromosome
		LRGAAPTLHFKG WLILDHIPS*				
GDEVAS EDDFQD GEDKRT SW	AST-B	MQLATLTATLLTLL AAAAAQDEGSGL AGAQAKRAGWSS MRGAWGKRTPDD TPEHGLQGSERN TIAKNGKHQLSER QRVAFRVRLTPDH TPITHTPPPTPPHT SPHTPPSTPPHMP PRWSQGVSEDKR NNNWSKFQGSW GKRGEEIQAAEDK RGGWNKFQGSW GKRGDEVASEDD FQDGEDKRTSWG KFQGSWGKRQDD LIHLQDLEDKRNN WSKFQGSWGKR AGWSSLQGAWG KRAWSNLQGAW GKRSPDDSEDIDD EALEDEELEVSPE TLARMVGAAPVK RGWVLWGKRPDY PAVSPRSTNWSS LRGAAPTLHFKG WLILDHIPS*	19-20	g30501	28	24
TSWGKF QGSW	AST-B	MQLATLTATLLTLL AAAAAQDEGSGL	19-20	g30501	28	24

Sequence	Family	Precursor	SP Length	Gene ID	Scaffold	Chromosome
		AGAQAKRAGWSS MRGAWGKRTPDD TPEHGLQGSERN TIAKNGKHQLSER QRVAFRVRLTPDH TPITHTPPPTPPHT SPHTPPSTPPHMP PRWSQGVSEDKR NNNWSKFQGSW GKRGEEIQAAEDK RGGWNKFQGSW GKRGDEVASEDD FQDGEDKRTSWG KFQGSWGKRQDD LIHLQDLEDKRNN WSKFQGSWGKR AGWSSLQGAWG KRAWSNLQGAW GKRSPDDSEDIDD EALEDEELEVSPE TLARMVGAAPVK RGWVLWGKRPDY PAVSPRSTNWSS LRGAAPTLHFKG WLILDHIPS*				
NNWSKF QGSW	AST-B	MQLATLTATLLTLL AAAAAQDEGSGL AGAQAKRAGWSS MRGAWGKRTPDD TPEHGLQGSERN TIAKNGKHQLSER	19-20	g30501	28	24

Sequence	Family	Precursor	SP Length	Gene ID	Scaffold	Chromosome
		QRVAFRVRLTPDH TPITHTPPPTPPHT SPHTPPSTPPHMP PRWSQGVSEDKR NNNWSKFQGSW GKRGEEIQAAEDK RGGWNKFQGSW GKRGDEVASEDD FQDGEDKRTSWG KFQGSWGKRQDD LIHLQDLEDKRNN WSKFQGSWGKR AGWSSLQGAWG KRAWSNLQGAW GKRSPDDSEDIDD EALEDEELEVSPE TLARMVGAAPVK RGWVLWGKRPDY PAVSPRSTNWSS LRGAAPTLHFKG WLILDHIPS*				
MVGAAP VKRGWV LW	AST-B	MQLATLTATLLTLL AAAAAQDEGSGL AGAQAKRAGWSS MRGAWGKRTPDD TPEHGLQGSERN TIAKNGKHQLSER QRVAFRVRLTPDH TPITHTPPPTPPHT SPHTPPSTPPHMP PRWSQGVSEDKR	19-20	g30501	28	24

Sequence	Family	Precursor	SP Length	Gene ID	Scaffold	Chromosome
		NNNWSKFQGSW GKRGEEIQAAEDK RGGWNKFQGSW GKRGDEVASEDD FQDGEDKRTSWG KFQGSWGKRQDD LIHLQDLEDKRNN WSKFQGSWGKR AGWSSLQGAWG KRAWSNLQGAW GKRSPDDSEDIDD EALEDEELEVSPE TLARMVGAAPVK RGWVLWGKRPDY PAVSPRSTNWSS LRGAAPTLHFKG WLILDHIPS*				
HQLSER QRVAF	AST-B	MQLATLTATLLTLL AAAAAQDEGSGL AGAQAKRAGWSS MRGAWGKRTPDD TPEHGLQGSERN TIAKNGKHQLSER QRVAFRVRLTPDH TPITHTPPPTPPHT SPHTPPSTPPHMP PRWSQGVSEDKR NNNWSKFQGSW GKRGEEIQAAEDK RGGWNKFQGSW GKRGDEVASEDD	19-20	g30501	28	24

Sequence	Family	Precursor	SP Length	Gene ID	Scaffold	Chromosome
		FQDGEDKRTSWG KFQGSWGKRQDD LIHLQDLEDKRNN WSKFQGSWGKR AGWSSLQGAWG KRAWSNLQGAW GKRSPDDSEDIDD EALEDEELEVSPE TLARMVGAAPVK RGWVLWGKRPDY PAVSPRSTNWSS LRGAAPTLHFKG WLILDHIPS*				
PDYPAVS PRSTNW SSLRGAA PTLHF	AST-B	MQLATLTATLLTLL AAAAAQDEGSGL AGAQAKRAGWSS MRGAWGKRTPDD TPEHGLQGSERN TIAKNGKHQLSER QRVAFRVRLTPDH TPITHTPPPTPPHT SPHTPPSTPPHMP PRWSQGVSEDKR NNNWSKFQGSW GKRGEEIQAAEDK RGGWNKFQGSW GKRGDEVASEDD FQDGEDKRTSWG KFQGSWGKRQDD LIHLQDLEDKRNN WSKFQGSWGKR	19-20	g30501	28	24

Sequence	Family	Precursor	SP Length	Gene ID	Scaffold	Chromosome
		AGWSSLQGAWG KRAWSNLQGAW GKRSPDDSEDIDD EALEDEELEVSPE TLARMVGAAPVK RGWVLWGKRPDY PAVSPRSTNWSS LRGAAPTLHFKG WLILDHIPS*				
GFYSQR Y	RYamide	MSRSLSPALFLLA TLITIAASQGFYSQ RYGKRGTDTRQIT ERSGFYANRYGR SQGIPEIKVRSSRF VGGSRYGKRSGA PALAEVALPVVVP ESEDSEVGASLLL GDSVVCLLVDMP DIYRCLKDQDTDL YSLAKKNSHQQC FS	21-22	g22592	49	2
APSGFLG MR	Tachykinin	MYVCMYVCMYLT PVYTHSMVRVWA WSVVVMVGVAAV AAAGGTEQVEES GREAARPRRAPS GFLGMRGKKEAP TPSLQADQANTED LLPALYQLDMPMR GKVTW	39-40	g44959	34	38

Table S1: Mature neuropeptides obtained from *C. borealis*. Abbreviations: B-type allatostatin (AST-B).

Sequence	Family	Precursor	SP Length	Gene ID	Scaffold	Chromosome
CFITNCPPG	Vasopressin	MQSGLAVTVV VTLLVGGAAS CFITNCPPGG KRSGGLMSTL GRARTCASCG PGLLGRCIGP DICCGARIGCF LGSRETRLCR TENMVPITCYN SDLKPCGRMQ EGRCGAPGLC CTEKV	20	03679	18	18
QLNFSPGW	Adipokinetic /RPCH	MISNSRAPTLG RRHTITEGDSP GRVRRSGVTL LVVALLVVTLM SSVSAQLNFS PGWGKRAAG ASGSNGGVGE AVSGLHPSVG GAPGGVVPPG SSSPGDSCGP IPVSAVMHIYR LIRSEAVRLVQ CQDEEYLG	48	05366	22	22
EAVANLAA RILKIVHAPH DAAAGVPH KRNSELINS LLGISALMN EA	PDH	MRSGVFVAVL VLVVFAALLTQ GQEIHVTERE AVANLAARILKI VHAPHDAAAG VPHKRNSELIN	22	14463	26	26

Sequence	Family	Precursor	SP Length	Gene ID	Scaffold	Chromosome
		SLLGISALMNE AGRR				
QEIHVTERE AVANLAARI LKIVHAPHD AAAGVPH	PDH1-like	MRSGVFVAVL VLVVFAALLTQ GQEIHVTERE AVANLAARILKI VHAPHDAAAG VPHKRNSELIN SLLGISALMNE AGRR	22	14463	26	26
EIHVTEREA VANLAARIL KIVHAPHDA AAGVPH	PDH1-like	MRSGVFVAVL VLVVFAALLTQ GQEIHVTERE AVANLAARILKI VHAPHDAAAG VPHKRNSELIN SLLGISALMNE AGRR	22	14463	26	26
QELHVPER EAVANLAA RILKIVHAPH DAAAGVPH	PDH1-like	MRSGVFVAVL VLVVFALLTQG QELHVPEREA VANLAARILKIV HAPHDAAAGV PHKRNSELINS LLGISALMNEA GRR	21	14465	26	26
NSELINSLL GISALMNEA	PDH1-like	MRSGVFVAVL VLVVFALLTQG QELHVPEREA VANLAARILKIV HAPHDAAAGV	21	14465	26	26

Sequence	Family	Precursor	SP Length	Gene ID	Scaffold	Chromosome
		PHKRNSELINSLLGISALMNEAGRR				
QELHVPEREAVANLAARILKIVHAPHDAAAGVPH	PDH1-like	MRSGVFVAVLVLVVFALLTQGQELHVPEREAVANLAARILKIVHAPHDAAAGVPHKRNSELINSLLGISALMNEAGRR	21	14465	26	26
EFTLRPTPTQAVANLAARILKIVHTPHDAAAGVPH	PDH2-like	MRSGVFVAVLVLVVLAALLTQGQQLHVPEREFTLRPTPTQAVANLAARILKIVHTPHDAAAGVPHKRNSELINSLLGISALMNEAGRR	22	14468	26	26
AVAAN	Ecdysis	MVGSRKVVVSALLVLSVALVLAVVLLPPSASAAVAANRKVSICIKNCGQCKKMYTDYFNGGLCGDFCLQTEGRFIPDCNRPDILIPFFLQRLE	32	18109	28	28

Sequence	Family	Precursor	SP Length	Gene ID	Scaffold	Chromosome
FIPDCNRPDILIPFFLQRLE	Ecdysis	MVGSRKVVVSALLVLSVALVLAVVLLPPSASAAVAANRKVSICIKNCGQCKKMYTDYFNGGLCGDFCLQTEGRFIPDCNRPDILIPFFLQRLE	32	18109	28	28
MYTDYFNGGLCGDFCLQTE[Amide]	Ecdysis	MVGSRKVVVSALLVLSVALVLAVVLLPPSASAAVAANRKVSICIKNCGQCKKMYTDYFNGGLCGDFCLQTEGRFIPDCNRPDILIPFFLQRLE	32	18109	28	28
NCGQCKKMYTDYFNGGLCGDFCLQTE	Ecdysis	MVGSRKVVVSALLVLSVALVLAVVLLPPSASAAVAANRKVSICIKNCGQCKKMYTDYFNGGLCGDFCLQTEGRFIPDCNRPDILIPFFLQRLE	32	18109	28	28
VSICIKNCGQCKKMYTDYFNGGLCGDFCLQTE	Ecdysis	MVGSRKVVVSALLVLSVALVLAVVLLPPSASAAVAANRKVSIC	32	18109	28	28

Sequence	Family	Precursor	SP Length	Gene ID	Scaffold	Chromosome
		IKNCGQCKKM YTDYFNGGLC GDFCLQTEGR FIPDCNRPDILI PFFLQRLE				
DSDESGSR Y	RYamide	MTAKVLLLVAL VAAAAAEGPT RSYNVPHDSS RSHESYESEE PKYSYNYNVK DDYGNDFGHE EKRDGDLTEG FYYNHLPDGR LQKVKYVVDG YSGFEADVSY EGEAHYDSGS YEYRRPVYRG GSRESGESRF GFGSGGSDES RFSFGSRGSD ESRFNSRSRE SGESRFGSDG SDESRFNSGS RESGESRFAF GGRGSDESKF NSDSRESGES RYGSGGSDES RFNSGSRGSG ESRFGFSRRG SDESRFNSGS RESGESRFRF	17	20419	27	27

Sequence	Family	Precursor	SP Length	Gene ID	Scaffold	Chromosome
		GRRGSDESRF NSGSRDSDES RYGYGGSDES RFDSGSRESG ESRFGFSRRG SDESRFNSGS RESGESRFRF GRRGSDESRF DSGSRDSDES GSRYGRGRS GESRSRGGS GEFRRFRSGG RSDEGFFLDQ YRPQISYSPAY DIRNRAKRRFI QSLMALDQVN RFGGLVKRISP YHLAAPPTTCI TTAGSDNDHQ GTAPSQLLRH PSVFCL				
ESGESRFR F	RYamide	MTAKVLLLVAL VAAAAAEGPT RSYNVPHDSS RSHESYESEE PKYSYNYNVK DDYGNDFGHE EKRDGDLTEG FYYNHLPDGR LQKVKYVVDG YSGFEADVSY	17	20419	27	27

Sequence	Family	Precursor	SP Length	Gene ID	Scaffold	Chromosome
		EGEAHYDSGS<br>YEYRRPVYRG<br>GSRESGESRF<br>GFGSGGSDES<br>RFSFGSRGSD<br>ESRFNSRSRE<br>SGESRFGSDG<br>SDESRFNSGS<br>RESGESRFAF<br>GGRGSDESKF<br>NSDSRESGES<br>RYGSGGSDES<br>RFNSGSRGSG<br>ESRFGFSRRG<br>SDESRFNSGS<br>RESGESRFRF<br>GRRGSDESRF<br>NSGSRDSDES<br>RYGYGGSDES<br>RFDSGSRESG<br>ESRFGFSRRG<br>SDESRFNSGS<br>RESGESRFRF<br>GRRGSDESRF<br>DSGSRDSDES<br>GSRYGRGRS<br>GESRSRGGS<br>GEFRRFRSGG<br>RSDEGFFLDQ<br>YRPQISYSPAY<br>DIRNRAKRRFI				

Sequence	Family	Precursor	SP Length	Gene ID	Scaffold	Chromosome
		QSLMALDQVN RFGGLVKRISP YHLAAPPTTCI TTAGSDNDHQ GTAPSQLLRH PSVFCL				

Table S2: Mature neuropeptides obtained from *C. sapidus*. Abbreviations: red pigment concentrating hormone (RPCH), pigment dispersing hormone (PDH).

Family	Precursor
Natalisin*	MDSVWLLLVMALAATLAATAQEVSPGEAGGSEGHSGAAAPWVGQRHARSLEAGGDASWLPVVQDEEVTQPVSGKGAGHGGTTFWVARGKKDAEGSLSYYWGPNQSLWGGEASHRGGPSMSNSPYVPLLQKSGWESNPSLWGKRDGHGPFWAARGKRPERDPFWVSRGRRDLAEAPPAPLHQWVAEDPAAQEHKDEQLWGGEIKREEGGPFWISRGKRPQPGSAASQLASLWAVRGRKSGADNTFWVARGKKETDPRGPFWAARGRRSGGEGGTGPYWIARGKKQDGTTPTGPYWIARGKKGDEDSVFWAARGKKDPPAWPTGRGRREDETHSFWIARGKKSGAITPRTAEKDNDDQGDNDEQHEEAMQKATDNYLKGFTTLAE
AST-B	MQLATLTATLLTLLAAAAAQDEGSGLAGAQAKRAGWSSMRGAWGKRTPDDTPEHGLQGSERNTIAKNGKHQLSERQRVAFRVRLTPDHTPITHTPPPTPPHTSPHTPPSTPPHMPPRWSQGVSEDKRNNNWSKFQGSWGKRGEEIQAAEDKRGGWNKFQGSWGKRGDEVASEDDFQDGEDKRTSWGKFQGSWGKRQDDLIHLQDLEDKRNNWSKFQGSWGKRAGWSSLQGAWGKRAWSNLQGAWGKRSPDDSEDIDDEALEDEELEVSPETLARMVGAAPVKRGWVLWGKRPDYPAVSPRSTNWSSLRGAAPTLHFKGWLILDHIPS
RYamide	MSRSLSPALFLLATLITIAASQGFYSQRYGKRGTDTRQITERSGFYANRYGRSQGIPEIKVRSSRFVGGSRYGKRSGAPALAEVALPVVVPESEDSEVGASLLLGDSVVCLLVDMPDIYRCLKDQDTDLYSLAKKNSHQQCFS
Tachykinin	MYVCMYVCMYLTPVYTHSMVRVWAWSVVVMVGVAAVAAAGGTEQVEESGREAARPRRAPSGFLGMRGKKEAPTPSLQADQANTEDLLPALYQLDMPMRGKVTW

Table S3: Annotated *C. borealis* neuropeptide precursors. Identified mature are underlined in green. Signal peptide denoted in red text. Putative dibasic cleavage sites denoted in blue text. *While this precursor encodes 15 mature natalisin peptides, peptide SGWESNPSLW includes the motif of a B-type allatostatin (AST-B) peptide.

Family	Precursor
Vasopressin	MQSGLAVTVVVTLLVGGAASCFITNCPPGGKRSGGLMSTLGRARTCASCGPGLLGRCIGPDICCGARIGCFLGSRETRLCRTENMVPITCYNSDLKPCGRMQEGRCGAPGLCCTEKV
RPCH	MISNSRAPTLGRRHTITEGDSPGRVRRSGVTLLVVALLVVTLMSSVSAQLNFSPGWGKRAAGASGSNGGVGEAVSGLHPSVGGAPGGVVPPGSSSPGDSCGPIPVSAVMHIYRLIRSEAVRLVQCQDEEYLG
PDH	MRSGVFVAVLVLVVFAALLTQGQEIHVTEREAVANLAARILKIVHAPHDAAAGVPHKRNSELINSLLGISALMNEAGRR
PDH	MRSGVFVAVLVLVVFALLTQGQELHVPEREAVANLAARILKIVHAPHDAAAGVPHKRNSELINSLLGISALMNEAGRR
PDH	MRSGVFVAVLVLVVLAALLTQGQQLHVPEREFTLRPTPTQAVANLAARILKIVHTPHDAAAGVPHKRNSELINSLLGISALMNEAGRR
Ecdysis	MVGSRKVVVSALLVLSVALVLAVVLLPPSASAAVAANRKVSICIKNCGQCKKMYTDYFNGGLCGDFCLQTEGRFIPDCNRPDILIPFFLQRLE
RYamide	MTAKVLLLVALVAAAAAEGPTRSYNVPHDSSRSHESYESEEPKYSYNYNVKDDYGNDFGHEEKRDGDLTEGFYYNHLPDGRLQKVKYVVDGYSGFEADVSYEGEAHYDSGSYEYRRPVYRGGSRESGESRFGFGSGGSDESRFSFGSRGSDESRFNSRSRESGESRFGSDGSDESRFNSGSRESGESRFAFGGRGSDESKFNSDSRESGESRYGSGGSDESRFNSGSRGSGESRFGFSRRGSDESRFNSGSRESGESRFRFGRRGSDESRFNSGSRDSDESRYGYGGSDESRFDSGSRESGESRFGFSRRGSDESRFNSGSRESGESRFRFGRRGSDESRFDSGSRDSDESGSRYGRGRSGESRSRGGSGEFRRFRSGGRSDEGFFLDQYRPQISYSPAYDIRNRAKRRFIQSLMALDQVNRFGGLVKRISPYHLAAPPTTCITTAGSDNDHQGTAPSQLLRHPSVFCL

Table S4: Annotated *C. sapidus* neuropeptide precursors. Identified mature are underlined in green. Signal peptide denoted in red text. Putative dibasic cleavage sites denoted in blue text. Abbreviations: red pigment concentrating hormone (RPCH), pigment dispersing hormone (PDH).

Family	Precursor
GSEFamide	MQVQGAVLVLLSASASAARPYLDQLPPRRTPTPYDFGYGVSVPATGDAKEHKE SQSLSGRTEGEYRWLQPNGLYR
Adipokinetic	MLHSDACSSGGGSRPHPARRPSLLYACCLASLWSSLALASVSRSPEGGRLLD ASPNGDVTLAWKSSNTSDSDNTTLSPVSSSSGDEKYKRTQFNGVLKYLPQAQ HDLLKFINSDSSRATRDAVDKAGNREDTQWLNMVPGTVERSDIPSAPPSPIGG DHSVLSKKVPLHIKHSNSRGSERLSPVATPLGNVAHSGARTLDRHEGVTGEGN GRVKGGSRHPLQQGPPRLPQPHPRTSTSDRMFLPDSTVSPLPVSSRPPAVFS SRPLKARQLNPLRIRLATHTKPSRLNFTPLAHPNHPNVSYVSHPSPSPSPSPYH TLPRLHNTANHTHTTTHPRPLNKPYHTSTGTPPRPYQHPYTGATPVNPSNLLLT HRTSNKATKPNTGKGIVGHQVPEAHKSKHTAPPGFFPSRRRLAAAAAAAAATT TPPPMYYTSPSTSTSTSTHKPAKTPVVPSSLLMTLADNSYRAPTKQQQRQQQP QQPQQQQQQKKQQQQQQQKKQPQQQLQLQPQQLQQLKQQHQQKYHQQQ QHEQPSLPNTPPASLPPPPPPQHTPQHSPAAFLPHTQITAAPPLSEANITCHFS HYLQLNKTAELDHEAESNITSRPSSSPVRPPLTPAGEDLLYVLRHVLGPLRQTS RVRPPTHGNLTGHFDAVGLARPLVSSQLPWTKPHSHYLGEDHLGHHKAPTPW GDYFTPHLNPINRLTPGKDSGAAHVLRLQGGGGAGHGASLVPAANPSSPVSLS TLLRLKAMNLPDSPPQHLSLMPLQQQQQHQHQQQHQQQQHNTNNNNNNNTS RVHSARHHSTSRSPRCPRGHHGAAGSGRARPPLCGLHGLGRQLGNVE
Neuroparsin	MNFAKIMLVCASLSVIVTSHTGGVLRGGHGAAGCVGTWPRRAGEAKTLQPAIP LDGMEDAAGELPRLRFDGTLRTVQEYKQHAFDRYLEIMKSQSEPSAFKDTQSR PRASRHIRRRPGMLMGSKGKDEVQEEEEEEAEFEAEVPRDPCLEGTPDFLCT VQLVPSGKRHIVSRKELEHNLREANETYSGFSLPFCCGGRASQMLLD
Corazonin	MNVVLVLAVVVGSCVCVAHPQYTHYSHHPRETSPNTTPPHTKGKEIQVPADTE NIQTEVKVQVDQSEIQHGKAHIVEEEDRKSHDIQRNTEARILDMNKRYPNRGKL RTLPGLVRLNQEGKPHINTTTTTTTSIQSKGCNFLCPKGDPNTVHL
RFamide	MLLLNICVFALTSAFVVVVKGGEARWKKIPNTPARPQRREVVVGGVRGPCTPP TPMWHLQENHRLKTARKRLESSWFRAEKTPASPLTVMGVSVSNVLNCGDQEE QVQRQRHEQEQEQQRQHIKRRRQFLQQEQQQQQQQQQQQQQHLLYHH LQQRPVDLGGSRLGRRRRRWQHWRAEAWHPRPLATHAAHAAHSPETTTLCC AVGESRR
RFamide	MRYSQVLQSQQFLVFLCLIVMAAAYAMSPVMNSSERYKTTLPLPATVQGHGIP RQEVNAPRDTQRSEFSIRLGMTVDLVTLLALAAAASAARPNYNFNYAVRDGDS

	RNDFGHEETRNLENTEGAFFVQLPDGRLQQVTYRVDGDSGFLADVSYKGDAR<br>FPDSDGSFESRERRVFSASRSGSAGRPSSTFSSASRSGSAGRPSSTFSSASS<br>SGSAGRPSSTFSSASSSGSAGRPSSTFFSASSSGSAGRPSSTFSSASRSGSA<br>GRPSTTFFSAPGSGSAARPSSTFFSRDSRESLEFPFASREDRFDSREDRFDSR<br>EDRFDSREDRFDSREDRFGSRFDSREDRFDSRFDSREDRFDSNESRGFFVAA<br>KNPFVRASSQRYSYA
RFamide	MLETLVVVVVVAVGVAGVGGSYLGGGGVGGAGVGGAGVGVVVMPAGPSRQY<br>TWAGQGHALYYPRPHPPRPLLIPRWVPDRDPTYTALWVPTTPPSAHAHHHAH<br>ARPAQRPSGFGPPKGNDLAPPTTVRVKSRPPVTRPSIKTPIPVSTHAHGTKGV<br>GVGGVILIAWASVWAWAWLCVGVFAAPHSPLRPRSYRWRGPGGVPDHAHNV<br>PRPPFFPRWLPDATPIYTSKWMPVPPTNTLRPHPHPPAGKLRPLTDKLLPPPH<br>PPTDKLRPPPRPPGSTAHTPSNHLGSSLITQSHTPALHQSESSLSFQSLPSKRH<br>ISRQSDNGLVTQSEQTLVDQSQEPLFPQSRPSLITQLRPVLITKSQPLDGPLLPLI<br>SQSEPFLITQSEHILVDQSDEMLLHQSQPSLITQSGPLMTQSAPIITQSEPLITQS<br>EQTLVDQSQEPNFYQSDRKEEDQDRKKEDQHRKEEDQHRKEEDQHRKEEDQ<br>HRKEDQDRKEEDQDRKEDQDRKEDQDREEDQDRKEDQDRK
AST-B	MKGLILLAATCVVVVVGEDTRGNFHGQGGLGGAHVGGAGVGGLVGGLVGGLV<br>GRPGQGSHGGRPGHGGHGGIHGGGQVGGIISGIVGGLGGHGGGHGGGHGG<br>GHGGGHGGGHGGGHGGGHGGGHGGGHGGGHLGGHGAGLVQGGKCGKSV<br>PTTQRYGCEFFVRDYRNQYVCDRNQTSLPVPCRPSYLHQELQWSSLTLRHRQ<br>RLQWLRQVLL
Adipokinetic	MVTMVVMVVVMVVVCGAGVESKNHLTSSSRETGRLLDQTNATTDKGNNAETS<br>EHRPSKECDRLRWRRWERVCLPQQECTKVRQQVWRERRVTWRGRNYLTLD<br>Y
RFamide	MTSYRNFQLVIPIRVLAAVWCVVAGGRGGATREASRLTPPPCITTPRHSPPPCA<br>LPPNTLHLPVSPPTLSTSPTLAPQPPVLAPLYKAVSLLRCGSRKSVNGCFLTEHI<br>SLKLVKIGGIRLSIRVERRWRRDAGNTSEEYGCEDKECDGNEDENEDRDEDAD<br>RDENLGTVWDAREPRKHVLASCLVETNCLLGARLVRREARQLRPRKHC
	MMASRWGKVGVVVLLTAALVMVESSSHCPSVCMCRWKNGKQTVECRNQQLA<br>SVPSVVDAETQVLDLNGNNLQNLPKYVFRRAGLINLQKIYLSNCNIGTIDNTAFA<br>QLTNLVELDLSDNLLTEVPAQAFSHVPALRDLQLGGNRLHKIHSYSFDHTPSLV<br>RLDLSFSGIKSVSTRAFENLSLLEKIELQGNQLTEVPVAVVRTLERVHEVDVHEN<br>NWICDCRALPLWRLLEVNNVPHPVSPSCGSPERIKGRAFHDLTERDFACPPHI

	RPNDRLVQGYAGENATIACPVDGQPPPEVMWYKGEAPVVNGSVIGLGPQRFY VITEGNRNVVSRLVITGAQETDSGLLRCVAINSAGSATANFTLAVTMRAATQAKL GKGHIAGISVGLGVLATIGIIVGLLILARTRTHSFPTPVKDSPTTPSSSEASPSEPN PVQKPPRLTDVNVSPATYRSSALGNPDVINEAERAVRAVNGHLPNGSVQEVSE GGDYTRVEGDSLYPSGLWPEEAASNEDADNPETSRVIHEHFNPGYMPNDSAY DGYGPLHSTPYRPEYGSQGEVDPQIYGYPADYGLPIPEAGNDPRARDWDMND LHGPQESVEPQADTYNSRQHIYESQQEVYNSDQNPKDSSQDLYGVTAGRPPV YGYRQEVFGSRENVAEGAESPQSPTQPLGERPWVPGSQAPPYARGGVAVLP PLPNGVANRIKARDSPDEGYQEGTEV
RFamide	MSRFLGIALLMVALSLTVTSQPFTQQDPSKHTGFIWNSRKIGNKKVATLTIRRMK SGIVAASPQWKVTTACTKKDRSRTITDTTTGRVHGRRSRRK

Table S5: Commonly found precursors in *C. borealis* tissues, with their peptides. Identified mature are underlined in green. Signal peptide denoted in red text. Putative dibasic cleavage sites denoted in blue text.

Family	Precursor
Insulin	MARVIEAAYLLLTLGAALAPGWADKPTDFHKGKSKYLCFYICFFVVDKFRVSRP<br>ETRQVTGYVLISHVDVKRIVLPSLQIIRGQTLFKVSVNDKSFALLVTLSKMHTLE<br>MPALRDSSRQRWRDQQLQPVPH
Insulin	MERVFITSLLAVWAADGATQSDSGSCPRPEDNPWCSCYDFNDGVFLECSVV<br>PLSRVATVLVQHRHPVKSLNIYDLESNLTSLPPALFVNSAGVSCLKISRSSLRD<br>VSESSFRGLQSSLHSLSIIHSHLAAIPQAALQRLPRLKTLDVEANNITELQSFSF<br>VNIKLSNLNMKGNGLKFISEYAFDGLEESLEELNLMNNKLKQLPIPALRRLSKLR<br>ILKSASNHISDVISDGYSRLPALQVLDLSSNRFTKLTSTSLSTMPALLSLSLCKN<br>HISEVAGDSFIQNSVLQRLCLSHNNIMILEPETFSYTLALQVIDLSHNHLHTISKG<br>LFSNLPELREVFLSFNNILKLQNDTFANSTQISFLYLHNNEIHSIESGAFANLEVL<br>FDLQISHNNLPEIPVGLFSSNRGLNTLSLDNNKITSLAGGTFSHLSELRELRLQN<br>NKLSRVEKSTFSPLPHLHELHLQDNLIEYVAKEAFSTLTELQNLNLKNNKLAQV<br>TDSLTRYPASLITLQYSKNQISTISTDALRGQNRLKILWLNDNNLTSMQEVLVHD<br>LSFLQELYLQNNNLGYIQDKCFHNMRKLKLLKLSNNNLQHISSLLFDGLTSLEV<br>LHLDHNGIREIEPRIFQRLVGLRHLDLSNNEILHVRRFMFEGEIPLKVLLLSSSGI<br>TDVDPYSFSSLNYLEELDLSKNSLAHLNSLTLSIPSIRVLKLGDNNFQIIEENSLY<br>GISNLNWLDMSGCGLFRLPPHLLSEALHLQHLNLARNNFTDLTSVFFRKLHNL<br>REVNISYNNFSHNVLNSVEGLTKLEVLILNHNPIEELRHPLNDLPSLHELHLSAT<br>NLWRLDRHILIRLKNLHTLDLSHNQLSDIPRGMLAGTSVSRLNLAENLFPQIPNT<br>IFQEGAPNLRSLNMSGNPMRRITDPLVAIGPPLPLLEDLEAIQTNLTTLTTYDLQ<br>QMPNLRYLNLSRASISRISPTSLRNLTHLTHLNLAYNRLQILPKERLRGLRSLTF<br>LNLTGNILKKLEPLPSASHSLKVLDASGNKLTGLAQSSFKHSECLEVLLLGANW<br>ITTIHPRTFLPLTTLRRLDLSHNYLEELSASPLEPLERSLEWLRLDGNPWRCEC<br>RLGQLWTWLQDHLSMVPDLSTLKCHLPEGFSGQPFLMLSSGMLCPQPLILQL<br>DAQGIQSQSLVLKWHASNQSSIQGYRVSFRETSEDGRVVGGMKTRSLTSSPN<br>NYRLTGLRPRTSYLVCLHGLTTSTNPTAIHHHHTTQYAPDLSSKCVQVKTDEPI<br>PAKIELSNRLAIMIGVSLGIAIFFIAGTIICCRQMCKDRREAAKAKLESNAGGQNY<br>HSYRQFSVQDNNDAADDSAPKEGQTEC
AST-B	MFSRGVLWLACCTCLVALVRVAECKGPCMKGLPAYKCSYRVDGQGRPRVLN<br>MKRLKRLFGSKGWTNVKKEPSPPRFFEVCCHGYVFYLIPPELAPPCLNGLWP<br>GECSWRPQGKGRLRMTNYEKMHQWERKGWKVVKKRSTGGPFREVCCSGK<br>VSYLAQQSSPTPKPGRKRAVEGKSVEGRKRSRERKRMSEKEPTPVKKPVSK

	KMPGEGRKKPATEKKKSVQGKKKSVPGKKTAPGKKITTLRKKTTPKKKPAAG KRKPATRKEKPAQGKKKSVPEKKLGSGKKKPIPRKKPVETSTPRKEASLQKKK KVKTSTPSALDKKKPVPSTKPVPVKTPSQRKKSLPQKRRGQGKKPAEGKKGA LEVKQEQKSRNTTSPKTETRIKTRPKTETRTKTRPKTETPTKTLPKDKITPIPPT VLPKVQLPPTTPPKNISVPVTLPKNVTVPSTPPKNITVSVTPPKNVTVPSTPPM NITAPTTFPNNETIPSFLSQNKTLPTPPTAPPKTEPAPTTLPKNVTAPSTLTKTK PPLTLTEKPSSPRNETSGKPKTALPRTSTASKTRSVQPETRRKRSTDGGQTEA LINETGQVQGEKDVKTALRKLEKEDKQGKMKGGHEFEHSHEEFSKDLENVVE EFHDEFAKEVKWRKHTGQMK
Insulin	MNTGPGCRAPLSLPGARAVWLWVWLWALALTHLGALADCPKVCECKWRDG KEVVTCRDAHFIDIPRGLDPSTQVLDLRHNNLKILPRDSFVYTGLVNLQKVWLN FCNLIKLEEGAFRMLNNVVELDLSNNYLQGVPSDALTDLPGLRVLRMAHNGLT VLPASAFLPVPDLVQLDLSHNTISEIESGALKSLASLGVLNLSGNKLTSLDVDEL KALMSLRVAHMDSNPWRCDCHLRPLSKWMQARNLAATIPPVCSFPRWLSGN NWQLLDEDEFVCAPVVTAVAPRVLAAEGENVSLACRVESEVETTITWLVGDA PLQNATEAQRYMVVEVFGPQHPVYISNLTISDVAPFDQGTYRCLAENRAGRG EVNLTLQVSHEVAEVRVATVDDSYYMKGGVVGGISIFAVVLLASCLLIYCKVRS TRRPRQEDDKLVTPQTSRGSDGGEHHTLTGYQVVPTSDMEEQSSSRRSQQI QPPWMLREASAAGKALEASGAESRRQESVDVPVTHPIAAPSTVSYVEIPKSG VGTVRTTLEDPTRWKDPLVYHSRDVLLHRLCGRSASQAATASTNGGHYPDLL DLPPQQQFQQQQEMQQHATYLPLHTPSPSYCTLPRTERAVKVTPRPGWKRG KLRRWRRQRRR
Insulin	MRKLWGWTLAALLWVVAAGVGPDYHSHDDCQVTSLPGTDLAALLCRLRTINS ELDATNFTVIPRRNTAKLRIECSDVLFFQSSLQNKSFVRLRELQELDIEYCKIGE VPREAFLGLTNLRNLTLRTYNTDWSAMSLKIASDAFREQRNLERLDLGDNNIW TLPPRLLCHLGNLKLLNLSRNKLQDVTVLSFSQAEHFCAPGLRHLDLSYNHLV SVPAFAFAALKNLQALNLSLNGISKLEDKALFGMYSLEVLDLSGNLLTALPPELF QENKRLTKLYIRNNSVSVLAPGLFTGLSLLLELELNDNQLTNTWVNSETFTDLL RLASLDLTNNKITRLDAATFRDLTNLQVLRLQHNMIETVSDYTFSGLFRLHTLVL SDNRIKELSDKTLAGLRGLQVLKLDHNEVFSVDSLALTNSTELQELQLSHNYLQ DVPKLVQSLTSLRTLDLSGNHVSVLTNSSFTDLNNLSALKLAANVVEVVSKTVF RALPALQVLDLSTNKIKTIENGAFVNNKHLEAVRLDNNLLTSVQGLFSELPNLQ WLNLSKNHLEMFDYAFIPRGLKYLDLRSNFINELGNYYEIESQLSLKIIDASFNK

	LSDISASSVPDSVEILFLNNNLISRVQPYAFFKKKNLTRVDLFANKIRNIDQNSLR LSLMNPSRDPPEFYLGGNPFECDCTMEWLQAINSLEETRQHPAVVDLDTINCR LMNNKGVIPLLEAQKLQFLCEYESHCFSLCHCCDFDACDCEMTCPSNCTCFH DESWAANIVDCSGAGYDTVPDRVPMDSSEVYLDGNELSSLSSHTFIGRKNLK VLYLNSSKVELIHNRTFNGLRLLEKLYLQHNMVKELKGYEFEHLTLLRELYLNS NELAFVHNATFLSLASLRILRLDDNRLKTLPVTYFRRNHNMHSLFLGNNPWQC ECESAAEVQTWLEGSAVVLRDPEQVQCVLNKTVEVTLQLTAFNVSVCTNESD TTTTIRHEAYLDYVFLPTVTLGAFAVLLTLTLIIFCNRNRMRVWVYAKYGVRLFY RSEYEGDTDKAFDAFVSYSSKDEVFVTQILAPELERGSPAYKLCLHYRDFPVG AYVTDTILSAVETSKRTILILSENFIKSEWRRFEFRSAHHEVLKDRRRRLIVILLG EVPRRDLDPDIRLYLKTNTYLKWGDNHFWEKLKFAMPDARRPSRSHQLHTLA AQPPPGPRPVPLHM
AST	MTAKVLLLVALVAAAAAEGPTRSYNVPHDSSRSHESYESEEPKYSYNYNVKD DYGNDFGHEEKRDGDLTEGFYYNHLPDGRLQKVKYVVDGYSGFEADVSYEG EAHYDSGSYEYRRPVYRGGSRESGESRFGFGSGGSDESRFSFGSRGSDES RFNSRSRESGESRFGSDGSDESRFNSGSRESGESRFAFGGRGSDESKFNSD SRESGESRYGSGGSDESRFNSGSRGSGESRFGFSRRGSDESRFNSGSRES GESRFRFGRRGSDESRFNSGSRDSDESRYGYGGSDESRFDSGSRESGESR FGFSRRGSDESRFNSGSRESGESRFRFGRRGSDESRFDSGSRDSDESGSRY GRGRSGESRSRGGSGEFRRFRSGGRSDEGFFLDQYRPQISYSPAYDIRNRA KRRFIQSLMALDQVNRFGGLVKRISPYHLAAPPTTCITTAGSDNDHQGTAPSQ LLRHPSVFCL
RFamide	VLLACAWVGVLTQTSKPRDDPQATTTVVPILRHINKLNGDGSYTYGFEAADGT FKLETRDALGNVKGKFGYTDNEGKLKVVEYAAGNGTGFETQSDVISSESTPPS FDTKLLSDLSSRRARRPQKQVKRQPSSSSPPPPPPPLSSSDFPQPSPQIKSRA HPTPHPQLPAEAESHDVFVPQFDPQPTTTFRPQRRQRPQRRRQSSRRHPTP QSEDSQPQPQPQHISRPRFRPQLPSHTDFEPQPHFGSQFPPQDDAFQPQHQ PQDTFRPQFLPGTQPQSAFVPDFTTQHDPESDPPPQPTFTPRFPSRPAFRPQ TPAARPPAFPPPVKEPEQFLRRKAEQDQDQDEEQQQIFPGPQFATNPFPQFG SRFPQADSGSQQFPPQPIVPKTPQFPRRPPGGSSFAGTNRFPLRKNEEEEDT PPSFPASPSQPSGVLPAGTFSISRPGRRLRPTPVSPKPQRVPSTQETPFFPQQ SQFSPQTPQFHGQEPSKFDDFDGDFAFGQNPSQFGQSPGQFGTPAPTFTRR PQFDRNPSPFDQGASHFGGQGVSSFGQGVSQPSQGASVFVQQSPQFGQGQ

	RKPQFGLGQGQGTPQFSPVAPQFGQGQGAPQFGQGQQAGLGQGKPQFGQ GQGAPQFGLGQGKPQFGQGQGAPQFGQGQGAPQFSPVAPHFGQGQRTPQ FGQGVQGVRGSPRPTPVPSQPDQVSALFGQGAHFDFGQNVSPFGQEAPHF GQDVSQPGQAPPAFDPVTPEPVQPKPQPGRGTSRFGQRRPTSSRGRPLTGR GTSPLDEKPDNSGQDTLSPNRGGARFGQNRPGFGGRPQTSQKTPQFGETVP PPTHKKPEFDKNPDEEEEKPVFGQQPAFPQPAFGQQPAFLGQPQFLPQPFA GQGFGFVTPQQLTPEQIKQIQQQGGRFSPQLIVPQQGFPVQGFQRFPVHPQQ RFGVPLTAGQPFLGTQPVFDPTQHSSFADDAKETDDTHDKQS

Table S6: Commonly found precursors in *C. sapidus* tissues, with their peptides. Identified mature are underlined in green. Signal peptide denoted in red text. Putative dibasic cleavage sites denoted in blue text.
